# Nomenclatural changes in *Centroptella* Braasch & Soldán, 1980 (Ephemeroptera, Baetidae)

**DOI:** 10.3897/zookeys.914.46652

**Published:** 2020-02-20

**Authors:** Nikita J. Kluge, Roman J. Godunko, Marek Svitok

**Affiliations:** 1 Department of Entomology, Saint Petersburg State University, Universitetskaya nab., 7/9, Saint Petersburg, 199034, Russia Saint Petersburg State University Saint Petersburg Russia; 2 Biology Centre of the Czech Academy of Sciences, Institute of Entomology, Branišovská 31, CZ-37005 České Budějovice, Czech Republic Biology Centre of the Czech Academy of Sciences České Budějovice Czech Republic; 3 Department of Invertebrate Zoology and Hydrobiology, University of Łódź, Banacha 12/16, 90237 Łódź, Poland University of Łódź Łódź Poland; 4 Faculty of Ecology and Environmental Sciences, Technical University in Zvolen, T. G. Masaryka 24, SK-96001 Zvolen, Slovakia Technical University in Zvolen Zvolen Slovakia; 5 Department of Ecosystem Biology, Faculty of Science, University of South Bohemia, Branišovská 1760, CZ-37005, České Budějovice, Czech Republic University of South Bohemia České Budějovice Czech Republic

**Keywords:** India, mayflies, new species, South Africa, systematics, Thailand, Vietnam

## Abstract

The genus *Centroptella* Braasch & Soldán, 1980 is accepted here in a wide sense, i.e., including *Chopralla* Waltz & McCafferty, 1987. This genus concept is similar to the concept of the genus *Bungona* Harker, 1957 proposed by Salles et al. (2016), but with the generic name *Centroptella* instead of *Bungona*. The type species of *Bungona*, *B. narilla* Harker, 1957, has an unknown systematic position; the neotype designation proposed by Suter and Pearson (2001) is invalid, being inconsistent with the International Code of Zoological Nomenclature; the species name *B. narilla* and the generic name *Bungona* are *nomina dubia*, so the name *Centroptella* is the senior name for the genus under consideration. The generic names *Chopralla* and *Crassolus* Salles, Gattolliat & Sartori, 2016 both are junior synonyms of *Centroptella* (**syn. nov.**). The subgenera *Bungona*, *Centroptella* and *Chopralla* proposed by Salles et al. (2016) are unnatural. The following new combinations are proposed: *Centroptella bintang* (Marle, Salles & Gattolliat, 2016) **comb. nov.**, *Centroptella bifida* (Shi & Tong, 2019) **comb. nov.**, *Centroptella fusina* (Tong & Dudgeon, 2003) **comb. nov.**, *Centroptella fustipalpus* (Lugo-Ortiz & McCafferty, 1998) **comb. nov.**, *Centroptella illiesi* (Lugo-Ortiz & McCafferty, 1998) **comb. nov.**, *Centroptella inzingae* (Crass, 1947) **comb. nov.**, *Centroptella papilionodes* (Marle, Salles & Gattolliat, 2016) **comb. nov.**, *Centroptella pontica* (Sroka, Godunko & Gattolliat, in Sroka et al. 2019) **comb. nov.**, *Centroptella ovata* (Shi & Tong, 2019) **comb. nov.**, *Centroptella quadrata* (Shi & Tong, 2019) **comb. nov.** and *Centroptella saxophila* (Agnew, 1961) **comb. nov.** The two Australian species, *C. fustipalpus* and *C. illiesi*, differ from each other in the shape of tergalii; corrections to the original description of *C. fustipalpus* are given based on re-examination of the holotype and paratypes; details of larval structures of *C. illiesi* are figured. Corrections to the former descriptions of the South African species *C. inzingae* and *C. saxophila* are given. Examination of type material led to the discovery that the original description of the Oriental species *Centroptella liebenauae* Soldán, Braasch & Muu, 1987 was based on two different species: the descriptions of imago and subimago belong to *Centroptella longisetosa* Braasch & Soldán, 1980 (the type species of *Centroptella*), and the description of larva belongs to a different species, which we describe here as *Centroptella ingridae***sp. nov.** The holotype of *C. liebenauae*, a larva, should be considered lost; based on the date of collection, it belonged to *C. longisetosa*; a set of larval exuviae with the same collecting data as the holotype, is designated as the neotype of *C. liebenauae*, and a new synonymy is established: *C. longisetosa* = *C. liebenauae***syn. nov.** The larvae originally assigned to *C. liebenauae* are placed to a new species *Centroptella ingridae***sp. nov.** belonging to the *inzingae*-*ingridae* species group; all stages of development of this species are described based on male and female imagines reared from larvae in Thailand and on the misidentified paratypes of *C. liebenauae* from Vietnam. *Centroptella longisetosa* is redescribed based on the single paratype from China, the neotype and paratypes of *C. liebenauae* from Vietnam, and additional material from India. Additional data on the holotype of *Centroptella colorata* Soldán, Braasch & Muu, 1987 are given.

## Introduction

Initially, the genus *Centroptella* Braasch & Soldán, 1980 was established for a single species, *C. longisetosa* Braasch & Soldán, 1980 described from China. Subsequently, other species of *Centroptella* were described from the Oriental Region, i.e., *C. ceylonensis* Müller-Liebenau, 1983, *C. similis* Müller-Liebenau, 1983 and *C. soldani* Müller-Liebenau, 1983 from Sri Lanka, *C. pusilla* Müller-Liebenau, 1984 from Borneo, *C. liebenauae* Soldán, Braasch & Muu, 1987 and *C. colorata* Soldán, Braasch & Muu, 1987 from Vietnam. Waltz and McCafferty (1987a) synonymized *Centroptella* with *Cloeodes* Traver, 1938, and at the same time proposed a new genus *Chopralla* Waltz & McCafferty, 1987, so that *longisetosa* [*Centroptella*] and *soldani* [*Centroptella*] were placed by them in the genus *Cloeodes*, and *ceylonensis* [*Centroptella*], *similis* [*Centroptella*] and *pusilla* [*Centroptella*] were placed in the genus *Chopralla*. In accordance with this classification, *Cloeodes fustipalpus* Lugo-Ortiz & McCafferty, 1998 and *Cloeodes illiesi* Lugo-Ortiz & McCafferty, 1998 were described from Australia, and *Chopralla fusina* Tong & Dudgeon, 2003 was described from Hong Kong; however, these three species have all the characters of *Centroptella*. Suter and Pearson (2001) stated that the Australian species *fustipalpus* [*Cloeodes*] and *illiesi* [*Cloeodes*] were identical to *Bungona narilla* Harker, 1957 and, thus, belonged to the genus *Bungona* Harker, 1957. Salles et al. (2016) reasonably stated that the East Hemisphere taxa *Centroptella* and *Chopralla* are closely related and different from the West Hemisphere taxon *Cloeodes*. At the same time, they accepted the interpretation of *Bungona narilla* proposed by Suter and Pearson (2001), and based on this, moved all *Centroptella* and *Chopralla* to the genus *Bungona* but treated these three taxa as subgenera. In accordance with this classification, Bungona (Centroptella) papilionodes Marle, Salles & Gattolliat, 2016 and Bungona (Chopralla) bintang Marle, Salles & Gattolliat, 2016 were described from Borneo; Bungona (Chopralla) pontica Sroka, Godunko & Gattolliat in Sroka et al. 2019 was described from Turkey; Bungona (Centroptella) ovata Shi & Tong, 2019, Bungona (Centroptella) quadrata Shi & Tong, 2019 and Bungona (Chopralla) bifida Shi & Tong, 2019 were described from China. Most descriptions were based on larvae only and, hence, lack some important taxonomic characters. Some of the species names mentioned above are synonyms, and some newly discovered (unpubl.) species of *Centroptella* from the Oriental and Afrotropical regions await description. Before these are described, however, the status of the formerly described taxa must be clarified.

## Material and methods

Imagines were reared from larvae in cages placed in natural flowing water and in containers with stagnant water. Part of material, including the holotype of *Centroptella ingridae* sp. nov., will be permanently deposited in the Russian Academy of Sciences, Zoological Institute, Zoological Museum (Saint Petersburg, Russia) (**ZIN**), but is temporarily located in the Department of Entomology of Saint Petersburg State University. The type specimens of *C. longisetosa*, *C. liebenauae* and *C. colorata* reported in this paper, which are deposited in the Institute of Entomology, Biology Centre, **CAS** (České Budějovice, Czech Republic) were temporarily moved to the Department of Entomology of Saint Petersburg State University during this study. The type specimens of *C. fustipalpus* reported in this paper, are deposited in the Purdue University Entomological Research Collection (West Lafayette, Indiana, USA); slides for these specimens were made by L.M. Jacobus using Euparal or BioQuip slide-mounting media. Other slides were made using Canada Balsam. In order to examine internal parts of penis and genital muscles of fresh specimens, genitalia were kept in hot water to dissolve non-translucent white inclusions; for this purpose, a glass with water and separated genitalia was placed on the cover of a desk-lamp. In the lists of material examined, the following arbitrary abbreviations are used: **L** – larva; **S** – subimago; **I** – imago; **L-S-I**♂ – male imago reared from larva, with larval and subimaginal exuviae; **L-S**♂ – male subimago reared from larva, with larval exuviae; **L/S**♂– male subimago extracted from mature larva.

The term “microlepide” is used according to Kluge and Novikova (2014), the terms “gonovectis”, “unistyliger” and “sigilla” according to Kluge and Novikova (2011); the term “protopteron” according to Kluge (2005), other terms according to Kluge (2004). The noun “blank” is used to describe an unpigmented or pale area.

For scanning electron microscopy (Figs 110–122), samples were gradually transferred to acetone, critical point dried and coated with gold by sputtering using a Baltec SCD050 Sputter Coater. Observations were taken on the scanning electron microscope (SEM) Jeol JSM 7401F at 4 kV in the Laboratory of Electron Microscopy, Institute of Parasitology, Biology Centre, CAS (České Budějovice, Czech Republic).

Other samples (Figs 81–82 and 126–128) were dried taken directly from alcohol, coated with gold and observed on the scanning microscope Jeol JCM-5000 (Neoscope) at 15 kV in the Centre for Molecular and Cell Technologies of St. Petersburg State University.

## Results

### Status of the generic name *Bungona*

Originally, the genus *Bungona* was established for a single species, *Bungona narilla* Harker, 1957, which was described from Coal and Candle Creek, Ku-ring-gai Chase National Park, Sydney (Australia). This species description was based on one male imago (holotype), one female subimago and one larva. The reason these three specimens were placed in one species was not reported. The description contains evident errors (tarsi of middle and hind legs were regarded to be 5-segmented, gonostyli were regarded to be 4-segmented, paraprocts were confused with the penis); the combination of other characters is different from any known species. The holotype and paratypes of *B. narilla* were stated to be housed in the British Museum (Natural History) (Harker 1957: 63; Suter and Pearson 2001: 247), but they disappeared and have not been reported among type specimens of this museum (Kimmins 1971).

Dean and Suter (1996) and Suter (1997) determined larvae they collected in Australia (Queensland, New South Wales, Victoria and Tasmania) as belonging to *B. narilla*, and based on this, they redefined the genus *Bungona*. However, these larvae differ significantly from the original description of *B. narilla* by the following characters. The apical segment of the labial palp is ovoid, without any concavities and points (Suter and Pearson 2001: fig. 22; Webb and Suter 2010: fig. 7); in contrast to this, the original description states that “the distal segment truncate”, and it is figured with the apex sharply pointed and the free margin deeply concave (Harker 1957: fig. 56). Larvae reported by Dean and Suter have the prostheca of the right mandible located close to the canines, with the distal branch running along the canines and the proximal branch arising from it under the right or blunt angle (Suter and Pearson 2001: figs 14, 15; Webb and Suter 2010: fig. 4). In contrast, the original description of *B. narilla* features a right mandible figured with both branches of the prostheca directed proximally and diverging at an acute angle (Harker 1957: fig. 53). Such prostheca form is found in various non-related taxa of Baetidae, including some species of *Centroptella* (Fig. 41), but not in the Australian species which Dean and Suter determined as *B. narilla*.

**Figures 1–15.**
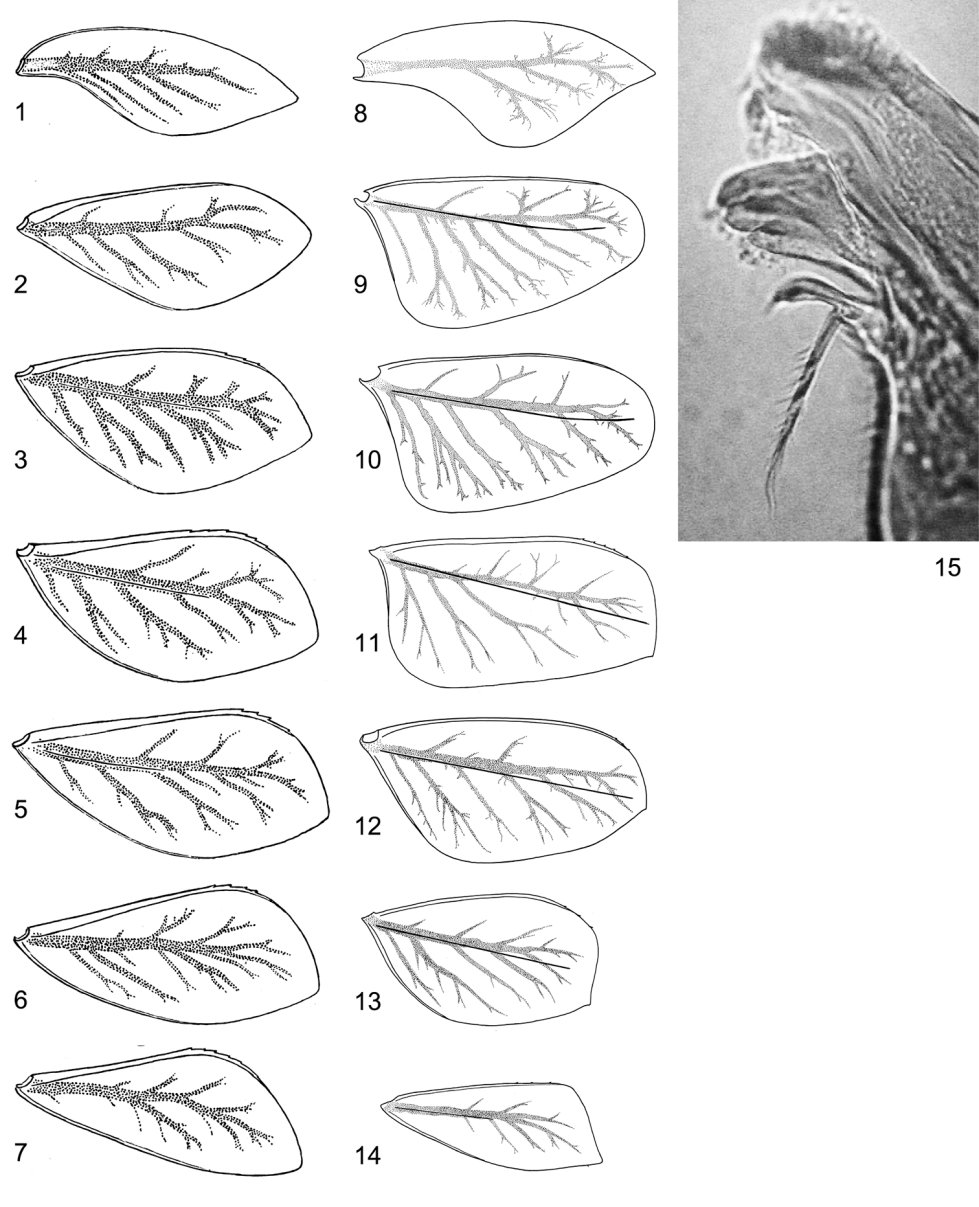
Australian *Centroptella*. **1–7***C. illiesi*, tergalii I–VII **8–14***C. fustipalpus* (paratype), tergalii I–VII **15***C. fustipalpus* (holotype), right mandible.

Lugo-Ortiz and McCafferty (1998) described larvae of two Australian species of *Centroptella* under the names *Cloeodes fustipalpus* Lugo-Ortiz & McCafferty, 1998 and *Cloeodes illiesi* Lugo-Ortiz & McCafferty, 1998. Suter and Pearson (2001) synonymized both these species names with *Bungona narilla*. According to the original description, *C. fustipalpus* differs from *C. illiesi* by having a non-bifid right prostheca and widened tergalii. As for the first character, “The specimen they illustrated and described as *C. fustipalpus* had a broken prostheca (subsequently confirmed by McCafferty, pers. comm.)” (Suter and Pearson 2001: 251). Our recent re-examination of the type material supports this conclusion: the proximal branch of the prostheca is not apparent on the right mandible of the paratype figured in the original description (Lugo-Ortiz and McCafferty 1998: fig. 3), but it is present on the holotype (Fig. 15). Concerning the shapes of tergalii, Suter and Pearson (2001) assumed that “this character may be influenced by age and environment”. However, descriptive data associated with various species of *Centroptella* from Asia and Africa (Kluge, unpublished) suggests that the shape of the tergalii is species-specific and constant at least among late larval instars. The holotype and all three paratypes of *C. fustipalpus* have tergalii of the 2^nd^ and next pairs sharply widened proximally (Figs 8–14), which is quite different from the lanceolate tergalii of *C. illiesi* (Figs 1–7). The tergalius of the paratype of *C. fustipalpus*, which was figured in the original description as “Gill 4” (Lugo-Ortiz and McCafferty 1998: fig. 8) actually belongs to the 2^nd^ pair (Fig. 9).

Suter and Pearson (2001) described male imagines ascribed to *B. narilla* based on specimens reared from larvae. In the same publication, they designated a neotype of *B. narilla*; this specimen is a non-reared male imago, collected from the Gara River, about 400 km north of the type locality. This choice of neotype (imago without associated larval exuviae) does not allow it to be compared with earlier described and reported forms, because most of them are known as larvae only. This neotype designation contradicts paragraphs 75.3.1, 75.3.5 and 75.3.6 of the International Code of Zoological Nomenclature (ICZN) and is invalid for the following three reasons.

1) “A neotype is validly designated when there is an exceptional need and only when that need is stated expressly and when the designation is published with the following particulars: 75.3.1. a statement that it is designated with the express purpose of clarifying the taxonomic status or the type locality of a nominal taxon ...”. All species taken into account in the publication, where the neotype of *B. narilla* was designated, i.e., *narilla* [*Bungona*], *fustipalpus* [*Cloeodes*] and *illiesi* [*Cloeodes*], were regarded as belonging to one species, and all their characters hitherto regarded as species-specific, were regarded as individual variability. In this situation, neotype designation is unnecessary, because it does not serve to clarify the taxonomic status of any nominal taxon.

2) “A neotype is validly designated when ... the designation is published with the following particulars: 75.3.5. evidence that the neotype is consistent with what is known of the former name-bearing type from the original description and from other sources”. There are no sources of knowledge about the holotype of *B. narilla* other than its original description, so the neotype can be compared only with the description given by Harker (1957). Besides characters common for all Baetidae, this description includes only details about the coloration of the abdomen, the proportions of hind leg segments, and the structure of genitalia. The following contradictions in characters between holotype and neotype were found:

In the holotype description, coloration is characterized as follows: “First two abdominal segments light brown, segments 3–7 yellow, the posterior segments light brown”; in the neotype description—coloration is characterized as follows: “abdominal segments 1–2 with central cream marking, 3 dark brown, 4 cream, 5–6 dark brown, 7–10 light brown”.

In the holotype description, hind leg proportions are characterized as follows: “tibia and tarsus equal in length, being about three-quarters length of femur. Tarsal segments of hind leg in decreasing order of length: 2, 3, 5, 4, 1 (fused with tibia)”; in the neotype description, hind leg proportions are characterized as follows: 1.00 : 0.74 : 0.09 : 0.18 : 0.10 : 0.08 : 0.15. That means, that the neotype has a femur/tibia/tarsus ratio of 1 : 0.75 : 0.6 (i.e., tibia and tarsus are not equal in length), and its tarsal segments in decreasing order of length are 2, 5, 3, 4, 1. The meaning of these numbers is unclear, because hind legs of all Baetidae have only 4 tarsal segments (including the first one, which is immovably fused with the tibia); but in any case, in the holotype the pen-penultimate segment is longer than the claw-bearing segment, while the neotype has the pen-penultimate segment shorter than the claw-bearing segment.

The drawing of gonostyli included with the holotype description (Harker 1957: fig. 50) does not resemble any known species, including the species described and figured in the neotype description. Words used to describe the holotype genitalia are as follows: “Forceps (fig. 50) 4-segmented; the second segment broad and short, arched on its inner surface, third segment much longer and also arched, distal segment small. Penis with a sharp spine distally (fig. 48); penis cover present”. Here unistyligers were interpreted as being the first segments, so 3-segmented gonostyli (“forceps”) were described as 4-segmented ones, and paraprocts were assumed to be the “penis”; thus, the only peculiar character is “penis cover present”. Judging by the figure in the original holotype description, its “penis cover” is a wide outgrowth of 9^th^ abdominal sternum, projected more distally than the unistyligers. In contrast to this, in the species to which the neotype belongs, the margin of the 9^th^ abdominal sternum between the unistyligers is straight and non-projected (Suter and Pearson 2001: fig. 4; Webb and Suter 2010: fig. 20).

3) “A neotype is validly designated when ... the designation is published with the following particulars: 75.3.6. evidence that the neotype came as nearly as practicable from the original type locality”. In the publication where this neotype was designated (Suter and Pearson 2001), a number of specimens were reported from localities much closer to the original type locality than the locality from which the neotype was collected. Thus, this neotype did not come from the nearest locality. The specimens determined as “*Bungona narilla*” and collected near the type locality, are larvae; but the paragraph 75.3.5 of the ICZN states: “a neotype may be based on a different sex or life stage, if necessary or desirable to secure stability of nomenclature”.

Later, Webb and Suter (2010) restricted the concept of *B. narilla*, which they continued to regard as conspecific with *fustipalpus* [*Cloeodes*], and they restored the species status of *Bungona illiesi* (Lugo-Ortiz & McCafferty, 1998).

The possibility to designate a new neotype after respective request to the International Commission of Zoological Nomenclature (according to Article 75.5 of the ICZN) can be a reasonable step for the rectification of this situation and taxonomic stability within the genus *Bungona*. Nevertheless, such a step should be taken only when new material of reared imaginal and larval specimens (preferably close to the type locality) is available. Despite considerable effort, such material is not available yet. Consequently, usage of the generic name *Bungona* is questionable and as such does not meet the requirements of the Article 23.9.1 of the ICZN.

The Australian Baetidae remain poorly known, with only 20 species described to date. Webb and Suter (2011) recognised 60 species, but most of these have not been formally described. We believe that the species originally described as *Bungona narilla* actually exists, and for this reason only this species (but not others) should bear this generic and specific name. The fact that specimens with characteristics of *B. narilla* have not been found in the vicinity of the type locality of *B. narilla* (Coal and Candle Creek) does not mean that *B. narilla* is a wrongly described *Centroptella*, because no specimen of *Centroptella* has been found in this place either (Suter and Pearson 2001: 250). It cannot be assumed as fact that the imago and larva Harker (1957) described under the name *B. narilla* really belong to one and the same species.

Given the inadequate nature of the original description, the loss of the type material, the improper assignment of a neotype, and the poorly documented diversity of related species in Australia, *Bungona narilla* (the type species of the genus *Bungona*) should be regarded as a *nomen dubium*. It then follows that the senior generic name for the species described below should be *Centroptella*.

#### Centroptella

Taxon classificationAnimaliaEphemeropteraBaetidae

Braasch & Soldán, 1980

461BA2D2-6D18-529E-95FD-84B0FB3156D6

Figs 1–15 , 16–18 , 19–39 , 40–48 , 49–52 , 53–59 , 60–76 , 77–80 , 81–82 , 83–89 , 90–93 , 94–109 , 110–115 , 116–122 , 123–128 , 129–132 , 133–137 , 138–143 , 144–148 , 149–152 , 153

= Chopralla Waltz & McCafferty, 1987: 182, syn. nov.
= Crassolus Salles, Gattolliat & Sartori, 2016: 104, syn. nov.

##### Type species.

*Centroptella longisetosa* Braasch & Soldán, 1980.

##### Systematic position and characters.

**Figures 16–18.**
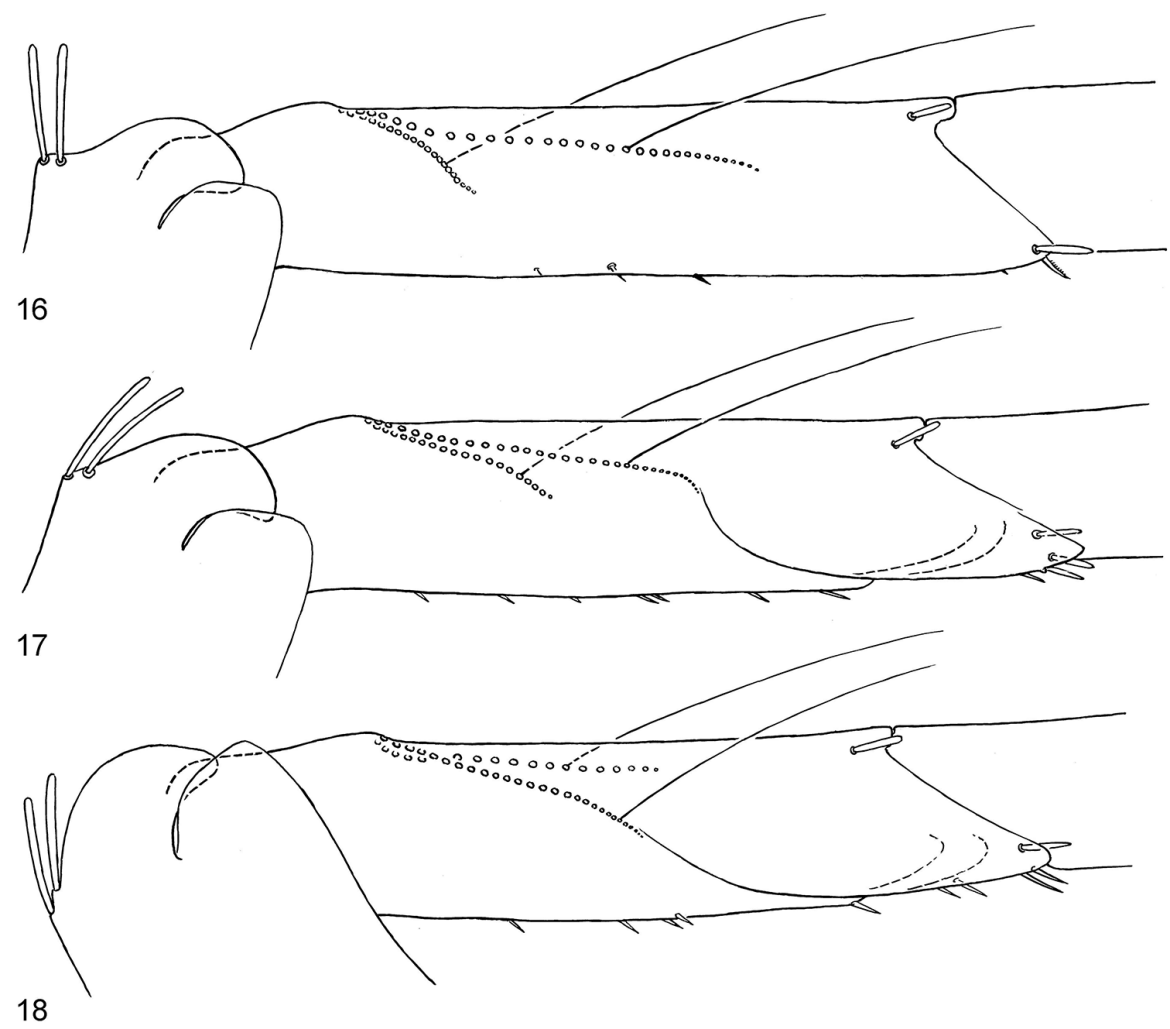
*Centroptella illiesi*, tibiae of fore, middle and hind legs, view from anterior side (bases of long setae shown both on anterior and posterior sides).

*Centroptella* is characterized by an unusual combination of characters: on one hand, it undoubtedly belongs to the holophyletic taxon Baetovectata Kluge & Novikova, 2011, based on (1) presence of two marginal intercalaries in each space of wing (Fig. 149), (2) narrow and arched gonovectes of the penis (Figs 77–80, 141–143, 146, 150152) and (3) medially inclined subimaginal gonostyli when they are developing under the larval cuticle (Figs 79, 148). The taxon Baetovectata belongs to the holophyletic taxon Anteropatellata Kluge, 1997, which is characterized by the presence of a patella-tibial suture on the forelegs of the larva and female imago and subimago. On the other hand, the leg structure of *Centroptella* does not conform with the characteristics of Anteropatellata. The larva of *Centroptella* has the structure of the tibia modified and different on each pair of legs, so that the patella-tibial suture is absent on forelegs and greatly shifted distally on the middle and hind legs; a row of long setae, which in some other taxa forms a transverse arc, in *Centroptella* is greatly stretched along the tibia, being different on the fore, middle and hind legs (Figs 16–18, 49–51, 90–92); the female imago and subimago of *Centroptella* has the usual leg structure, with the patella-tibial suture not shifted distally, but without patella-tibial suture on forelegs. This leg structure is characteristic of the plesiomorphon Protopatellata Kluge & Novikova, 2011 and has striking similarity with the Afrotropical taxon *Potamocloeon* Gillies, 1990 (= *Maliqua* Lugo-Ortiz & McCafferty, 1997), which undoubtedly belongs to Protopatellata and has no features of Baetovectata (Kluge 2019). The Neotropical genus *Cloeodes* Traver, 1938, which some authors have confused with *Centroptella* (see above), has none of these features, and its larval and imaginal leg structure is typical for Anteropatellata (Kluge 2017).

Besides this paradoxical combination of baetovectatan and protopatellatan characters, *Centroptella* has an evident autapomorphy: secondary swimming setae on the outer sides of the larval cerci in the distal part of the cercus have oval transverse bases and form a regular row (Figs 59, 129–131); in this respect, they resemble the primary swimming setae on the inner side of the cercus (Fig. 132), but they are smaller and less densely arranged.

Another peculiar character of *Centroptella* is the presence of a pair of spaced transverse rows of long bifurcate setae on certain abdominal sterna of the larva (Figs 58, 117–119); in different species these setal rows are present on sterna II–VI or on part of them, at least on sterna IV–V. Identical setal rows are found in a few other, non-related taxa (e.g., *Potamocloeon* Gillies, 1990 and *Cloeodes* Traver, 1938).

### Status of the genus-group name *Chopralla*

Waltz and McCafferty (1987a) divided the Old World genus *Centroptella* into two parts, one of which (including the type species of *Centroptella*) they united with the New World genus *Cloeodes* Traver, 1938, and for another one established a new genus *Chopralla* with the type species *Centroptella ceylonensis* Müller-Liebenau, 1983. The genus *Chopralla* was separated from *Cloeodes = Centroptella* “by the absence of ventral tufts of setae on abdominal segments 2–6, the apically rounded gills (versus broadly pointed in *Cloeodes* species), the peculiar claw structure (unlike edentate claws of *Cloeodes*), and the possession of long, fine tibial seam setae (not present in *Cloeodes* species)”. Among these four characters, only the difference in claw structure exists in reality, while the other three characters were reported erroneously (Kluge 2017). At the same time, the Old World species attributed by Waltz and McCafferty to *Cloeodes*, are closely related to the species placed by them in *Chopralla*, being distant from the New World species of *Cloeodes*. Because of this, Salles et al. (2016) united these Old World species in one genus, leaving only the New World species in the genus *Cloeodes*. At the same time, they changed the generic name *Centroptella* to the name *Bungona*, which they regarded to be its senior synonym (see above), so the generic name *Chopralla* was regarded to be a junior synonym of *Bungona*. Here we recognise the generic name *Centroptella* as a valid one, thus a new formal generic synonymy is established: *Centroptella* = *Chopralla*, syn. nov. If the genus *Centroptella* is divided into subgenera, one of these subgenera should bear the subgeneric name *Chopralla* (see below).

### Status of the genus-group name *Crassolus*

The genus *Crassolus* Salles, Gattolliat & Sartori, 2016 was established for a single South African species *Crassolus inzingae* (Crass, 1947), which was originally described in the genus *Pseudocloeon* (Crass 1947) and subsequently placed in the genus *Baetis* (Gillies 1994) and then in the genus *Cloeodes* (Waltz and McCafferty 1994). The species *Pseudocloeon saxophilum* Agnew, 1961 which was originally described from the Western Cape Province, was regarded to be a junior synonym of the species *inzingae* [*Pseudocloeon*], which was originally described from Natal (Waltz and McCafferty 1994).

Examination of reared material of *saxophilum* [*Pseudocloeon*] collected in the Western Cape Province in 2019 (Figs 151, 152), reveals that this species has all the characters of *Centroptella* and is closely related to *C. ingridae* sp. nov. described below.

Salles et al. (2016) did not provide direct comparison of the new genus *Crassolus* with the genus under the name “*Bungona*”. In their phylogenetic schemes (Salles et al. 2016: figs 1, 2), the genus *Crassolus* is opposed to the whole branch comprising the genera “*Bungona*” (actually *Centroptella*) and *Cloeodes*. It seems, however, that the existence of the branch (indicated as Node 72) was not based on autapomorphies. Node 72 was characterised by five apomorphies under the numbers 3, 9, 40, 42 and 48, none of which separates it from *Crassolus*:

Character “3” (distance between prostheca and incisors of right mandible) was said to have increased from 0.00 (ancestral condition reported for *Crassolus inzingae*) to 0.04 (Node 72). Actually, according to the matrix of characters (Appendix S3), among the species attributed to “*Bungona*”, this characters varies from 0.00 to 0.26. The condition “0.00” was reported for the larvae determined as “Bungona (Chopralla) liebenauae” and actually belongs to the new species *Centroptella ingridae* sp. nov. described below (Fig. 145).

Character “9” (length of fore femur / distance between base of fore femur and base of most distal setae of fore femur) was said to have increased from 0.92 (ancestral condition reported for *Crassolus inzingae*) to 0.95 (Node 72). Actually, according to the matrix of characters, among the species attributed to “*Bungona*”, this characters varies from 0.92 (in three species included in the matrix) to 1.00.

Character “40” (slender process on prostheca of right mandible) was said to have changed from “0=absent” (ancestral condition reported for *Crassolus inzingae*) to “1=present” (Node 72). Actually, according to the matrix of characters, this process is absent in the larvae determined as “Bungona (Chopralla) liebenauae” and actually belongs to the new species *Centroptella ingridae* sp. nov. described below (Fig. 145).

Characters “42” and “48” are “setae between prostheca and mola of right mandible” and “long setae between prostheca and mola of left mandible”. The both characters were said to have changed from “1=present” (ancestral condition) to “0=absent” (in the Node 72). In the matrix of characters (Salles et al. 2016: Appendix S3), the condition “1=present” was reported for *Crassolus inzingae*, in contrast to all species included at Node 72 (including all species attributed to “*Bungona*”), for which the condition “0=absent” was reported. Vice verse, the diagnosis of the genus *Crassolus* (Salles et al. 2016: p. 105) included the words: “absence of long setae between prostheca and mola of both mandibles”, while the diagnosis of the genus *Bungona* (ibid., p. 100) included the words: “spine-like setae between prostheca and mola of right mandible present”. On the drawings (Salles et al. 2016: figs 4C–F) the right mandibles of *Crassolus inzingae* and Bungona (Chopralla) ceylonensisis were shown without setae between prostheca and mola, but the right mandibles of Bungona (Bungona) narilla and Bungona (Centroptella) soldani were shown with these setae. Actually, in all species of *Centroptella* setae between the prostheca and the mola vary individually from very small to absent (Figs 15, 40, 41, 144, 145).

The monotypic genus *Crassolus* was said to be characterized by six apomorphies under the numbers 0, 2, 5, 7, 20 and 22 (Salles et al. 2016: p. 96, fig. 1 and Appendix S2). Actually, all these characters are found among the species attributed by these authors to “*Bungona*”: Character “0” (length of body) was reported as 6.0 mm for *Crassolus* and as 2.5–6.2 mm for “*Bungona*”; acording to the original description, in *Crassolus inzingae* it varies as 4.5–6.0 mm (Crass 1947). Character “2” (angle of subtriangular process of left mandible) was reported as 2.38 for *Crassolus* and as 2.35–2.70 for “*Bungona*”. Character “5” (length of fore femur/ length of fore tibia and tarsus combined) was reported as 0.85 for *Crassolus* and as 0.80–1.05 for “*Bungona*”. Character “7” (length of setae on outer margin of fore femur / width of fore femur) was reported as 0.22 for *Crassolus* and 0.29–0.63 for “*Bungona*”. Character “20” (length / width of fore wing) was reported as 2.78 for *Crassolus* and as 2.39 and 2.44 for two species of “*Bungona*”with known imagines; however, in the species whose larvae were determined as “Bungona (Chopralla) liebenauae” (which is described here as *Centroptella ingridae* sp. nov.) this proportion is 2.94 (Fig. 149). Character 22 (number of spaces in RS sector of fore wing with marginal intercalary veins) was reported as 8 for *Crassolus* and as 8 and 0 for two species of “*Bungona*” (possibly, misprints).

The type species of *Crassolus* is closely related to *Centroptella ingridae* sp. nov. described below; these species have many common characters, including peculiar halberd-like tips of gonovectes not found in other taxa (Figs 137–143, 146, 150–152). In the original description of *Pseudocloeon inzingae*, the gonovectes were neither described, nor figured (Crass 1947: fig. 9g). In the redescription of this species under the name *Cloeodes inzingae*, gonovectes were adequately figured behind unistyligers, but with small hooks instead of the halberd-like structures (Waltz and McCafferty 1994: fig. 6). In the subsequent redescription of this species under the name *Crassolus inzingae*, the halberd-like structures were drawn, but the proximal borders of unistyligers (located externally) were draws by interrupted lines as internal structures, probably being confused with the gonovectes (Salles et al. 2016: fig. 9D). Here the genitalia of lectotype are figured based on the photo, to show the correct position of gonovectes and outlines of unistyligers (Fig. 150).

Salles et al. (2016: appendix S3) believed that larva of *Crassolus inzingae* had no denticles on claws, in contrast to *Chopralla*. According to the original description, its “claw without denticulations” (Crass 1947: p. 62 and fig. 9e). Subsequently this character never had been checked, neither for *inzingae* [*Pseudocloeon*], nor for *saxophilum* [*Pseudocloeon*] (Agnew 1961; Waltz and McCafferty 1994). Actually, the type specimens of both species have a few small denticles by the sides of the claw, similar to that of *Centroptella ingridae* sp. nov. (Figs 126, 127) (Helen Barber James, personal communication).

Based on the above, the following generic synonymy is suggested: *Centroptella* = *Crassolus*, syn. nov. Within the genus *Centroptella*, several Asian and African species, including *C. inzingae* and *C. ingridae* sp. nov., constitute a natural species group, characterized by the halberd-like tips of the gonovectes and other common characters in imaginal and larval structure.

### Subgeneric classification of *Centroptella*

Salles et al. (2016) divided the genus *Centroptella* (under the name “*Bungona*”) into three subgenera, two Asian subgenera *Chopralla* and *Centroptella*, and one Australian subgenus under the name “*Bungona*”.

Among them, the subgenus Chopralla was an artificial group, because one of its species belongs to the *inzinagae*-*ingridae* group, while another species of the *inzinagae*-*ingridae* group was placed in a separate genus *Crassolus* (see above).

Two other subgenera, *Centroptella* and “*Bungona*” had not been separated one from another by any currently recognized characters.

According to the diagnosis of the subgenus Bungona, “Dorsal surface of labrum with two setae on anterolateral corner” (character “1”). In contrast, according to the original descriptions by Lugo-Ortiz and McCafferty (1998), there are 6–8 setae in *fustipalpus* [*Cloeodes*] and 3–4 setae in *illiesi* [*Cloeodes*].

According to the diagnoses of the subgenera *Bungona* and *Centroptella*, they differ by the distance between the prostheca and mola of the right mandible (character “3”). However, the structure and position of the right prostheca is the same in the larva determined as “*Bungona narilla*” and in *Centroptella soldani* (Salles et al. 2016: figs 4C, D).

According to the diagnosis of the subgenus Centroptella, it has “few setae on outer margin of fore femur (around six)”, in contrast to 10 in *Bungona* (character “14”). However, according to original descriptions by Lugo-Ortiz and McCafferty (1998), there are 5–8 setae in *fustipalpus* [*Cloeodes*] and 5–7 setae in *C. illiesi* [*Cloeodes*].

According to the diagnosis of the subgenus Bungona, it has “angle of row of long setae on posterior surface of fore tibia around 60°”, in contrast to 30° in *Centroptella* (character “13”). Actually this angle is around 30° both in the species attributed to *Bungona* (Fig. 16) and in the type species of *Centroptella* (Fig. 49), in contrast to around 60° in the species attributed to *Chopralla* (Fig. 90).

Thus, the subgeneric classification proposed by Salles et al. (2016) is inconsistent. In the present paper we accept the genus *Centroptella* (= *Chopralla* = *Crassolus*) without dividing it into subgenera.

### Composition of the genus *Centroptella*

Considering the factors discussed above, the genus *Centroptella* should be accepted as comprising the following nominal species (alphabetically): *Centroptella bifida* (Shi & Tong, 2019) comb. nov.; *Centroptella bintang* (Marle, Salles & Gattolliat, 2016) comb. nov.; *Centroptella ceylonensis* Müller-Liebenau, 1983; *Centroptella colorata* Soldán, Braasch & Muu, 1987; *Centroptella fusina* (Tong & Dudgeon, 2003) comb. nov.; *Centroptella fustipalpus* (Lugo-Ortiz & McCafferty, 1998) comb. nov.; *Centroptella illiesi* (Lugo-Ortiz & McCafferty, 1998) comb. nov.; *Centroptella inzingae* Crass, 1947 comb. nov.; *Centroptella liebenauae* Soldán, Braasch & Muu, 1987; *Centroptella longisetosa* Braasch & Soldán, 1980; *Centroptella ovata* (Shi & Tong, 2019) comb. nov.; *Centroptella papilionodes* (Marle, Salles & Gattolliat, 2016) comb. nov.; *Centroptella pontica* (Sroka, Godunko & Gattolliat) (in Sroka et al. 2019) comb. nov.; *Centroptella pusilla* Müller-Liebenau, 1984; *Centroptella quadrata* (Shi & Tong, 2019) comb. nov.; *Centroptella saxophila* Agnew, 1961 comb. nov., *Centroptella similis* Müller-Liebenau, 1983; *Centroptella soldani* Müller-Liebenau, 1983. Below, a new synonymy *C. longisetosa* = *C. liebenauae* is established, and a new species, *C. ingridae* sp. nov. is described. In subsequent publications, some other synonyms will be proposed and several new species of *Centroptella* from the Oriental and Afrotropical regions will be described.

### Type specimens of *Centroptella liebenauae*

Under the name “*Centroptella liebenauae*”, Soldán et al. (1987) described two different species, one of which was described as larva, and the other as male and female imagines. These descriptions were based on specimens collected at the same place (Suoi Bac Stream near Tam-Dao Mountain in Vietnam), but at different times: larvae described as “*Centroptella liebenauae*” were collected in autumn 1985, and imagines described as “*Centroptella liebenauae*” were reared from larvae in spring 1982. Therefore, in order to determine the correct application of the name “*Centroptella liebenauae*”, it is necessary to examine the holotype. However, during the course of this research, we discovered some problems associated with this holotype, which we detail below. The following specimens and labels were examined by us; they are deposited in the collection of the Institute of Entomology (BC CAS) in České Budějovice, Czech Republic:

(1) Mature male larva with labels: “VIETNAM, Vinn Phu Prov., Soui Bac – Tam Dao, 10–16.X.1985 T. Soldán”, “*Centroptella liebenauae* T. Soldán det. 1985” and “HOLOTYPE”;

(2) 46 larvae with labels: “VIETNAM, Vinn Phu Prov., Suoi Bac Stream, Tam-Dao, 10–16.10.1984 T. Soldán”, “*Centroptella liebenauae* T. Soldán det. 1985” and “PARATYPES”; many of these larvae are late instars, and some are ready to moult to subimago;

(3) tube with 3 male imagines (one without genitalia), 1 male subimago, 1 female imago, 1 male larval exuviae and 1 abdomen of female subimago extracted from mature larva, with labels: “VIETNAM, stream, Tam-Dao 60 km NW of Hanoi, 23–25.5.1982 T. Soldán”, “*Centroptella*, T. Soldán det. 1982” and “PARATYPE”; now larval exuviae, parts of one male imago and parts of male subimago are mounted on slides in Canadian balsam. The larval exuviae are designated as the neotype of *Centroptella liebenauae* (see below).

All specimens in tubes (1) and (2) belong to one and the same species, which is described below as *C. ingridae* sp. nov., and all specimens in tube (3) belong to a single, different species, which is *C. longisetosa*.

According to the original description, the holotype is a larva collected 23–25.V.1982 together with an additional 18 larvae and 5 reared winged insects (three male imagines, one female imago and one male subimago), while larvae collected 10–16.X.1984 and 17.X.1984 are paratypes. This means that the larva labelled as “holotype” was actually collected 10–16.X.1984 (not 16.X.1985), and is not the holotype, but a paratype. We speculate that the true holotype (i.e., the specimen designated as the holotype in the original publication) is mixed among the 18 other larvae collected on the same dates (23–25.V.1982) and now cannot be recognized among them.

Judging by the list of specimens examined in the original description, among the specimens contained in the tube (3), the male imago without genitalia is “paratype No. 1”, the single female imago is “paratype No. 2” and the single male subimago is “paratype No. 3”; the single set of male larval exuviae belongs to one of four males in this tube. The location of the 19 larvae from this series (including the true holotype) is unknown. The lost holotype was an intact larva; no structures were mounted on any slide, so its details were not examined, and the authors of the original description could not have known to which of the two species it belonged.

Sometimes larvae of different species of *Centroptella* can be collected at the same place (NJK; unpublished data). In the case of the larvae of the two species described under the name “*Centroptella liebenauae*”, they differ in size, shape and coloration; these differences are easily visible if they lie together, but such differences can be overlooked if they are examined separately. The fact that the authors of the original description did not notice these differences indicates that they never saw larvae of these two species side-by-side. Judging by the fact that all 47 larvae collected 10–16.X.1985 belong to *C. ingridae* sp. nov., and all five reared imagines and subimagines collected 23–25.V.1982 belong to *C. longisetosa*, we assume that all larvae collected 23–25.V.1982, including the lost holotype of *C. liebenauae*, also belong to *C. longisetosa*. It is well known, that even in unimpaired rivers composition of mayfly communities varies considerably over time (e.g., Svitok 2006; Leunda et al. 2009) and has well-expressed seasonality in Oriental streams (e.g., Dudgeon 1984); it follows that the conditions in the Suoi Bac Stream are likely to be different in May and in October, and between the years 1982 and 1985.

### Neotype designation for *Centroptella liebenauae*

Complete set of last instar male larval exuviae (Figs 26–32, 47, 52–59, 76) with the geographical label “VIETNAM, stream, Tam-Dao 60 km NW of Hanoi, 23–25.5.1982 T. Soldán” is designated here as the **neotype** of *Centroptella liebenauae*. All part of these exuviae are mounted on slide in Canada Balsam, except for abdomen and tergalii, which are mounted on the same object glass in dry condition, under a separate cover glass. These exuviae belong to one of the four males—three imagines (Fig. 80) and one subimago, but is unclear to which because the rearing was not individual. Each of these three male imagines and one male subimago are labelled now as “possibly from neotype”. The neotype (male larval exuviae) and all four-winged male specimens, each of which can belong to the neotype, as well as female imago in the same tube, will be permanently deposited in the Institute of Entomology (BC CAS) in České Budějovice, Czech Republic.

### New synonymy caused by neotype designation

Based on this neotype designation, we propose a new synonym: *Centroptella longisetosa* = *Centroptella liebenauae* syn. nov.; another species, described under the name “*Centroptella liebenauae*”, is a new species, and it is described here under the name *C. ingridae* sp. nov. (see below).

### Reasons for the neotype designation

According to the Article 75.3 of the International Code of Zoological Nomenclature, a neotype is validly designated when there is an exceptional need and only when that need is stated expressly and when the designation is published with the particulars listed in the paragraphs 75.3.1–75.3.7. In the present case, such exceptional need is present, because usage of the same name *Centroptella liebenauae* for two distant species causes confusion; all particulars required in the paragraphs 75.3.1–75.3.7 are published here as the following:

“75.3.1. a statement that it is designated with the express purpose of clarifying the taxonomic status ...”. This purpose is to choose, which of two different species originally described under the name *Centroptella liebenauae*, should bear this name.

“75.3.2. a statement of the characters that the author regards as differentiating from other taxa the nominal species-group taxon for which the neotype is designated ...” These characters are give below, in the discription of *C. longisetosa*.

“75.3.3. data and description sufficient to ensure recognition of the specimen designated”. These data are given above.

“75.3.4. the authors’ reasons for believing the name-bearing type specimen(s) (i.e., holotype, or lectotype, or all syntypes, or prior neotype) to be lost or destroyed, and the steps that had been taken to trace it or them”. The steps that had been taken to trace the holotype, are reported above; the lost holotype is an intact larva, whose individual features have never been reported or figured; even its sex is unknown. If this specimen is found in future, it will be impossible to prove that it is the holotype designated in the original publication, as it could be any other specimen among 19 lost larval specimens, which have one and the same geographical label.

“75.3.5. evidence that the neotype is consistent with what is known of the former name-bearing type from the original description and from other sources ...”. The original description contains characters and figures of two different species, *C. longisetosa* and *C. ingridae* sp. nov. Based on the original description, we know that the former name-bearing type was collected in spring 1982, and analyzing the collection we know that all specimens collected at that time belong to *C. longisetosa*. Designating the neotype from specimens collected in autumn 1985 and belonging to a species different from the holotype, would clearly violate this paragraph.

“75.3.6. evidence that the neotype came as nearly as practicable from the original type locality ...”. The neotype comes from the type locality and has the same date of collecting as the holotype.

“75.3.7. a statement that the neotype is, or immediately upon publication has become, the property of a recognized scientific or educational institution, cited by name, that maintains a research collection, with proper facilities for preserving name-bearing types, and that makes them accessible for study”. This institution is the Institute of Entomology (BC CAS) in České Budějovice, Czech Republic, where this specimen was deposited recently.

Besides these formal rules, the Code requires maintainance of prevailing usage of the taxa names which can be done only under plenary power of the Commission (paragraph 75.6). In this case, referring to prevailing usage is impossible, because there are only six publications where the species name *liebenauae* [*Centroptella*] has been mentioned (Soldán et al. 1987; Soldán 1991; Tong and Dudgeon 2003; Salles et al. 2016; Sroka et al. 2019; Shi and Tong 2019). Among them, reports of larvae under this name given by Soldán (1991) and by Shi and Tong (2019) do not contain original taxonomic conclusions. Soldán et al. (1987) and Salles et al. (2016) applied this name both to *C. longisetosa* and *C. ingridae* sp. nov. at the same time; the phylogenetic reconstruction proposed by Salles et al. (2016) was based on a matrix of characters which contained larval characters of *C. ingridae* sp. nov. and imaginal characters of *C. longisetosa* under the common name “Bungona (Chopralla) liebenauae”. As a result, the classification based on this phylogenetic reconstruction was unnatural, and the closely related species *C. inzingae* and *C. ingridae* sp. nov. would be placed in different genera (see above, Status of the genus-group name *Crassolus*). Tong and Dudgeon 2003 used imaginal characters of what they called “*Chopralla liebenauae*” to confirm species identity of the newly described species *Chopralla fusina*; in this case the name “*Chopralla liebenauae*” was applied to *Centroptella longisetosa*. Sroka et al. (2019), vice verse, used larval characters of what they called “Bungona (Chopralla) liebenauae” to confirm species identity of the newly described species Bungona (Chopralla) pontica; in this case the name “*Chopralla liebenauae*” was applied to *Centroptella ingridae* sp. nov. Thus, the name *liebenauae* [*Centroptella*] has been equally often applied to both *C. longisetosa* and *C. ingridae* sp. nov.

Our choice of the neotype, being the single one is consistent with the Code. Moreover, the single one provides the both considered species with monosemantic valid names, because the non-monosemantic (equivocal) name *C. liebenauae* becomes invalid. The designation of a neotype and the new synonymy proposed here will stop further confusion caused by inaccurate descriptions of two different species under the one name, *C. liebenauae*.

### Type specimens of *Centroptella longisetosa*

The type species of the genus *Centroptella*, *C. longisetosa*, was originally described based on larvae from China. According to the original description (Braasch and Soldán 1980), the holotype of *C. longisetosa* is “Larve (Präparat in Kanadabalsam mit Tellosolve). VR China, Liu Chui, Fluß im Kuj Fon Shan; 11.XII.1959, leg. I. Hrdý”. Besides the holotype, six larvae were reported from the same locality. The place of deposition of the type material was reported as “Holotypus und 5 Paratypen in der Coll. Soldán, Praha; 1 Paratypus in der Coll. Braasch, Potsdam”.

One of us (RJG) examined the mayfly collection deposited in the Institute of Entomology (BC CAS) and could not find the slide with the holotype of *C. longisetosa*. Consultations with Tomáš Soldán also did not clarify the fate of the holotype. Instead, there is a tube with larva in alcohol with labels: “CHINA, Liu Chiu, Kuj Fon Shan Mt., stream, 11.12.1959, leg. Ivan Hrdý”, “*Centroptella longisetosa* T. Soldán det. 1980” and “HOLOTYPE”. Judging from the original description, this intact larva is not the holotype, but one of five paratypes deposited in the Soldán collection. Another paratype was reported by Waltz and McCafferty (1987a, 1987b) and by Tong et al. (2003), as a male larva in alcohol, “deposited Purdue Entomological Research Collection, originally from paratypes in the collection of T. Soldán”. This specimen is present in the collection and has been assigned number “PERC-0063355” (Luke M. Jacobus, personal communication). Waltz and McCafferty (1987a) reported the collection data as: “Peoples Republic of China, Liu Chui, Kuj Fon Shan River, 11-12-1959, I. Hrdy.” The collection label, however, says: “China: Liu Chui river at Kuj Fon Shan 11.12.59 leg. I Hrdý”.

Waltz and McCafferty (1987b) reported that the specimen in the Purdue University Entomological Research Collection “had absorbed the ink used in labelling and is entirely black and devoid of pattern”. The specimen deposited in the Entomological Institute is also black, but this cannot be a result of ink absorption; possibly, it is a preservation artefact from vinegar acid having been added to alcohol at some point.

#### Centroptella longisetosa

Taxon classificationAnimaliaEphemeropteraBaetidae

Braasch & Soldán, 1980

05AA0EB1-B63A-5978-A812-D879736A154C

Figures 19–32 , 40–48 , 49–52 , 53–59 , 60–76 , 77–80 , 81–82

Centroptella longisetosa Braasch & Soldán, 1980: 123 (larva)
Cloeodes longisetosus : Waltz and McCafferty 1987a: 179 (lava); 1987b: 201, 206 (larva); Tong et al. 2003: 669 (larva, ♂ & ♀ imago)
Bungona (Centroptella) longisetosa : Salles et al. 2016: 104, figs 6B, C, K, 7F, 9C (larva, ♂ imago); Shi and Tong 2019: 572, figs 1–5 (larva)
Centroptella liebenauae Soldán, Braasch & Muu, 1987: 342 (partim: ♂ & ♀ imagines, ♂ subimago; non larva), syn. nov.
Chopralla liebenauae : Tong and Dudgeon 2003: 19 (comparison of ♂ imago)
Bungona (Centroptella) liebenauae : Salles et al. 2016: Appendix S3 (partim: imaginal characters 20–30 and 122–131)

##### Material examined.

**Paratypes of *Centroptella longisetosa*** (deposited in the Institute of Entomology, BC CAS, České Budějovice and Purdue University Entomological Research Collection, West Lafayette, Indiana, USA): mature female larva with labels: “CHINA, Liu Chiu, Kuj Fon Shan Mt., stream, 11.12.1959, leg. Ivan Hrdý”, “*Centroptella longisetosa* T. Soldán det. 1980”, “HOLOTYPE” (now parts of this specimen are mounted on 2 slides, eggs mounted for SEM; one middle larval leg of another specimen, in the same tube (now treated by alkali and mounted on separate slide); one larva, Peoples Republic of China, Liu Chui, Kuj Fon Shan River, 11-12-1959, I. Hrdy, PERC-0063355.

**Neotype and paratypes of *Centroptella liebenauae*** (deposited in the collection of the Institute of Entomology, BC CAS, České Budějovice): one tube containing: 3 male imagines (one without genitalia), 1 male subimago, 1 female imago, 1 male larval exuviae (neotype) and 1 abdomen of female subimago extracted from mature larva, with labels: “VIETNAM, stream, Tam-Dao 60 km NW of Hanoi, 23–25.5.1982 T. Soldán”, “*Centroptella*, T. Soldán det. 1982” and “PARATYPE”; now larval exuviae (neotype) and parts of male imago and male subimago (one of which possibly was reared from the neotype) are mounted on slides.

##### Additional material.

INDIA, Tamilnadu, Tirunelveli District, Courtallam, Chittar River near Peraruvi (= Main Falls), 3–7.II.2013, coll. N. Kluge & L. Sheyko: 3 L-S-I♂, 1 L-S-I♀, 1 S-I♀, 1 L/S♂, 1 L/S♀, 1 larva of penultimate instar.

##### Descriptions.

***Larva*.***Cuticular coloration.* Head mostly brown (Fig. 55); specimens from India mostly colourless, but with frons brown. Pronotum and mesonotum brown with diffuse lighter and darker areas (Fig. 53). Thoracic pleura and metanotum partly brown, partly colourless; sterna colourless (Fig. 57). Forecoxa colourless; middle and hind coxa laterally brown, medially colourless; femur of each leg light, with large, diffuse, brown macula on posterior surface; tibia of each leg light at middle, at base and apex diffusely tinged with brown; tarsus of each leg proximally brown, with gradation to colourless distally; claws colourless (Fig. 54). Abdominal terga brown with lighter areas; some terga with light medioanterior sigilla; terga IV and VIII lighter than others (Fig. 57). Caudalii colourless (Fig. 56).

*Shape and setation*. Frontal suture short, nearly semicircular (Fig. 55). Labrum equally wide at base and middle, with pair of submedian long setae, 3–4 pairs of sublateral long setae and pair of long setae between submedian and sublateral ones (Fig. 43). Prostheca of left mandible with 3 blunt processes and 3 pointed processes (Fig. 40). Prostheca of right mandible directed medially or medially-proximally, bifurcate, with branches diverging under acute angle and longest branch directed proximally (Fig. 41). Maxillary canines and distal dentiseta stout; distal dentiseta widened, with apex somewhat hooked toward canines (Fig. 42). Maxillary palp in specimens from China and Vietnam short, either 2-segmented, or indistinctly 3-segmented (Figs 46, 47); in specimens from India long and distinctly 3-segmented (Fig. 48). Labium with glossae and paraglossae subequal, both narrowed apically (Figs 44–45). Glossa ventrally with irregularly arranged setae in proximal part and 4–6 setae forming ventro-median row. Paraglossa with latero-apical setae forming one regular row and few (2–4) setae just dorsal of it; with 4–6 setae in ventro-median row; with 3 setae in dorso-median row. Distal segment of labial palp widened apically (Fig. 45).

All thoracic terga without protuberances. Metanotum with vestiges of hind protoptera (Fig. 54; Tong et al. 2003: fig. 7). Femora of all legs equal, tibia and tarsus on foreleg longest, on hind leg shortest; on foreleg tarsus longer than tibia, on middle and hind legs tarsus shorter than tibia (Fig. 54); in paratype length of femur / tibia / tarsus / claw of foreleg (mm) 0.75 : 0.48 : 0.54 : 0.13; on middle leg 0.75 : 0.47 : 0.44 : 0.13; on hind leg 0.75 : 0.44 : 0.41 : 0.13. Femur parallel-sided; outer margin straight or slightly concave, apically with blunt-angled projection bearing two subapical setae; inner margin slightly convex (Fig. 54). Outer side of femur with row of 5–7 long blunt setae and 2 subapical setae of same form (Fig. 49). Inner-dorsal side of forefemur with few stout setae, these setae being half length of setae on dorsal side; middle and hind femora with minute setae only. Foreleg without patella-tibial suture, middle and hind legs with patella-tibial suture greatly shifted to apex of tibia. Posterior arm of U-shaped row of long setae on fore- and middle tibiae oblique and directed more longitudinally than transversely (Figs 49–50); on hind leg longitudinally (Fig. 51). Inner margin of tibia and tarsus with irregular, small, stout, pointed setae. Outer-apical seta of tibia blunt and elongate (Figs 49–51). Dorsal side of each tarsus with long, fine setae, situated irregularly and partly forming two longitudinal rows. Claw without denticles.

Scales on abdominal terga and sterna numerous, short, semicircular, colourless and delicate (Figs 52, 60–76). Posterior margin of abdominal tergum I smooth, without denticles; posterior margins of terga II–VI partly without denticles, partly with short semicircular and triangular denticles; terga VII–IX with longer, triangular denticles (Figs 60–68); on tergum IX denticles located behind pair of submedian setae, smaller and denser than others (but row of denticles not interrupted at this place) (Fig. 68). Posterior margin of tergum X with even row of small, narrow, pointed denticles (Fig. 52). Posterior margins of abdominal sterna I–III smooth; posterior margin of sternum IV with few, minute denticles (Fig. 70); posterior margins of sterna V–VIII with regular pointed denticles, increasing in length from sternum V to sternum VIII (Figs 71–74). Posterior margin of sternum IX in female convex, with even row of triangular denticles (Fig. 75), in male with narrow and dense denticles between protogonostyli and by sides of them (Fig. 76). Each sternum IV–VI with pair of regular, transverse rows of long, fine, bifurcate setae with spaced sockets; other sterna either without such setae, or with few, smaller setae irregularly situated (Figs 57–58). Paraprocts without anterior median apodeme, with many small, pointed denticles on posterior margin, with scales as on sterna and terga (Fig. 52).

**Figures 19–39.**
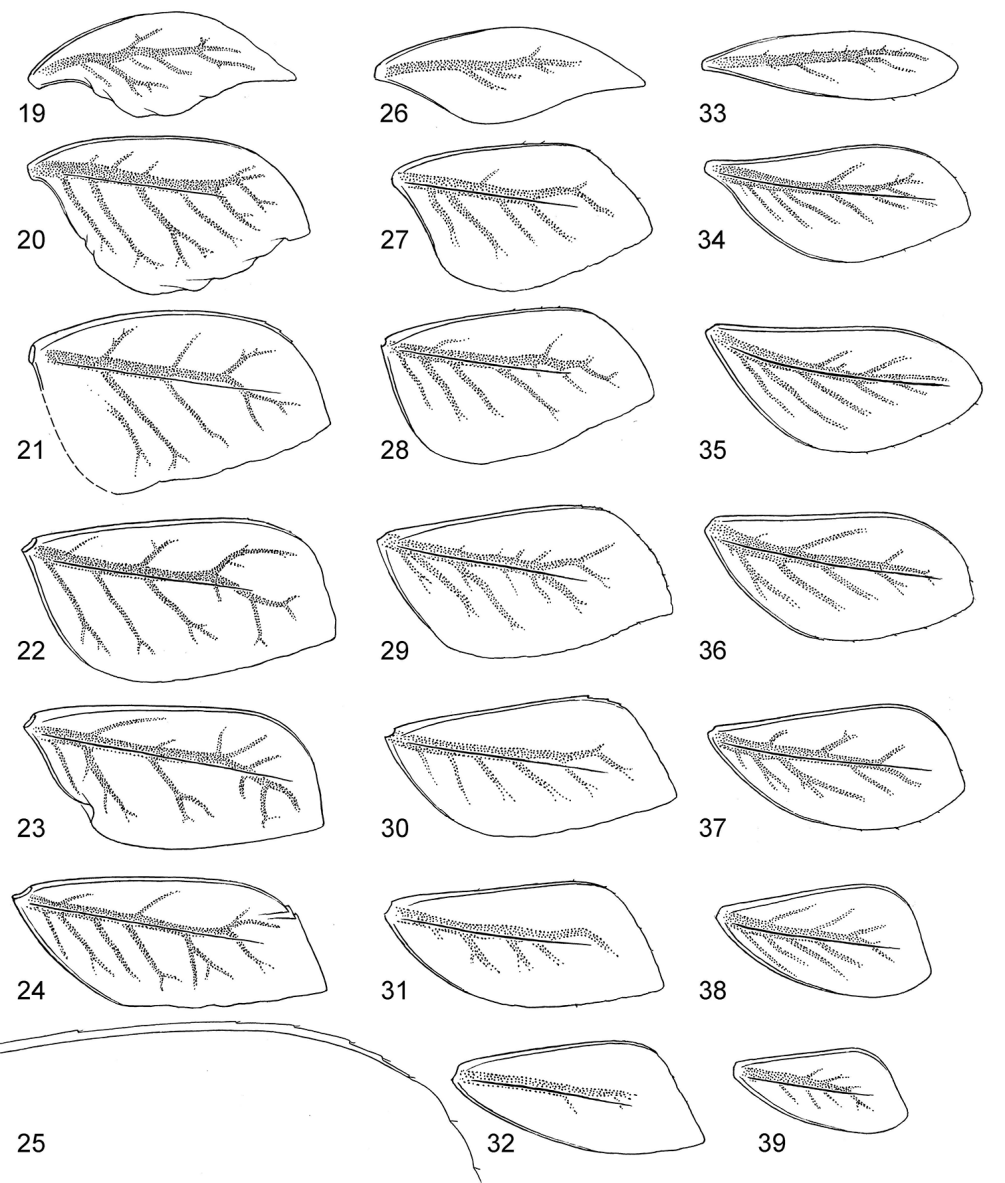
Asian *Centroptella*. **19–32***C. longisetosa*: (**19–24**) tergalii I–VI of paratype of *C. longisetosa* (**25**) the same, apex costal rib of tergalius IV (**26–32)** tergalii I–VII of neotype of *C. liebenauae* (actual *C. longisetosa*) **33–39***C. ingridae*, tergalii I–VII of sp. nov.

Tergalii apically pointed and sharply differentiated as follows: tergalius I lanceolate, slightly bent, widened at midlength, with apex stretched and narrowly pointed (Figs 19, 26); tergalius II especially wide, widest at proximal half, with anal margin more convex than costal margin (Figs 20, 27); tergalii III–VI with gradation of shapes (Figs 21–24, 28–31); tergalius VII narrow, widest at distal half, with costal margin more convex than anal margin (Fig. 32). Each tergalius II–VII, besides costal and anal ribs, with straight and narrow middle rib, located on dorsal surface on background of main trachea. Costal margin with poorly expressed serration (Fig. 25); anal margin without serration; outer margin free of ribs, slightly notched, with small seta in each notch. Lateral side of each cercus with several long, pointed denticles on each 4^th^ segment (Figs 56, 59). Each cercus, besides regular row of primary swimming setae on inner side, with smaller and thinner secondary swimming setae on outer margin; on most part of cercus secondary swimming setae with wide transverse oval bases, forming regular row (Fig. 59); on proximal 1/5 of cercus secondary swimming setae with small round bases and situated irregularly.

*Male genitalia* (examined in Indian specimen): In last larval instar, developing subimaginal gonostyli folded under larval cuticle in peculiar pose, with 3^rd^ segments bent medially-proximally (Fig. 79).

**Figures 40–48.**
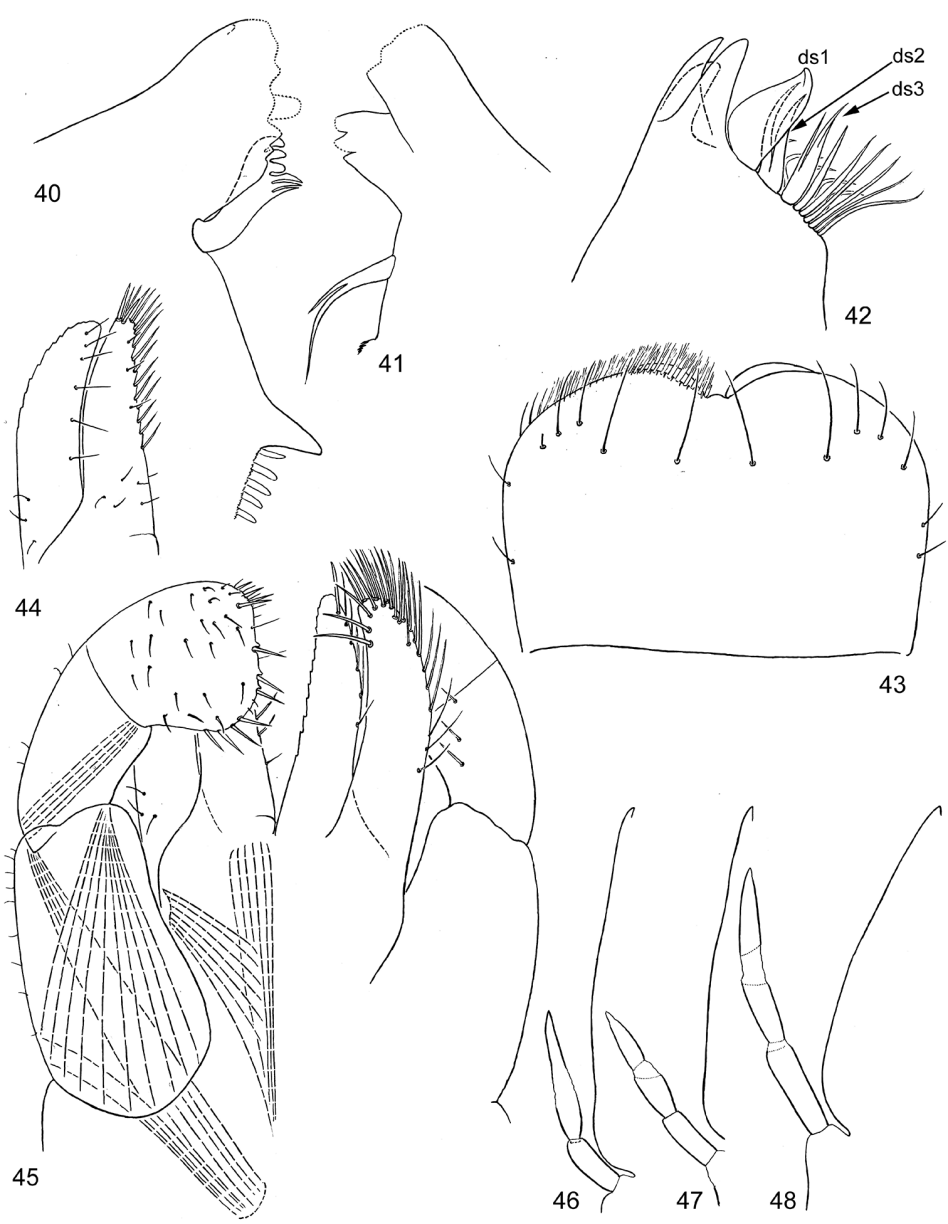
*Centroptella longisetosa*. **40–46** paratype of *C. longisetosa*: (**40**, **41**) left and right mandibles (**42**) apex of maxilla (**ds1**, **ds2**, **ds3** dentisetae) (**43**) labrum (**44**) glossa and paraglossa (ventral view) (**45**) labium (at left ventral view, at right dorsal view; muscles shown by interrupted lines) (**46**) maxillary palp **47** the same, neotype of *C. liebenauae* (actually *C. longisetosa*) **48** the same, specimen from India.

**Figures 49–52.**
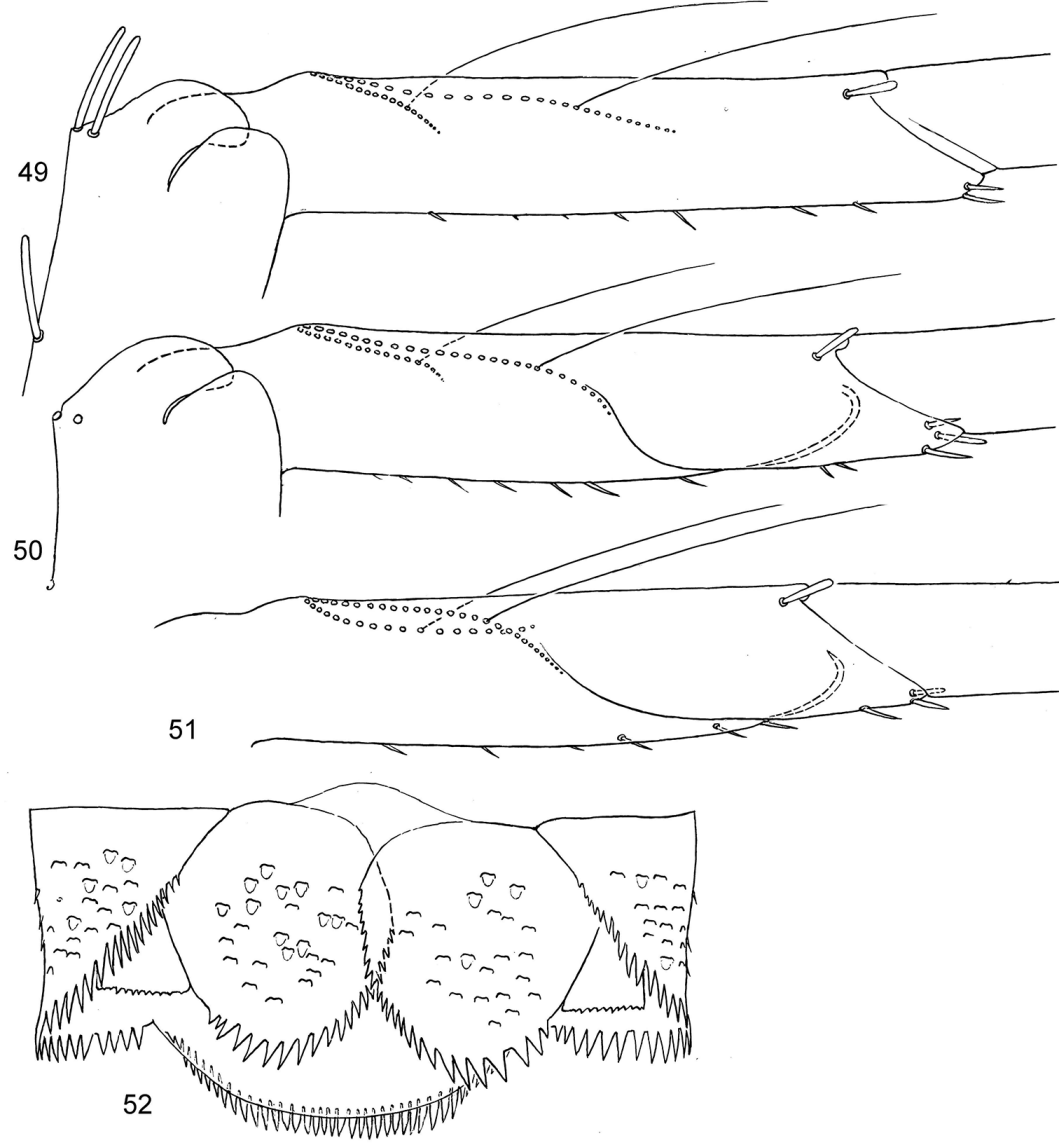
*Centroptella longisetosa*. **49–51** paratype of *C. longisetosa*: tibiae of fore, middle and hind legs, view from anterior side (bases of long setae shown both on anterior and posterior sides) **52** neotype of *C. liebenauae* (actually *C. longisetosa*): tenth abdominal segment without caudalii, ventral view.

**Figures 53–59.**
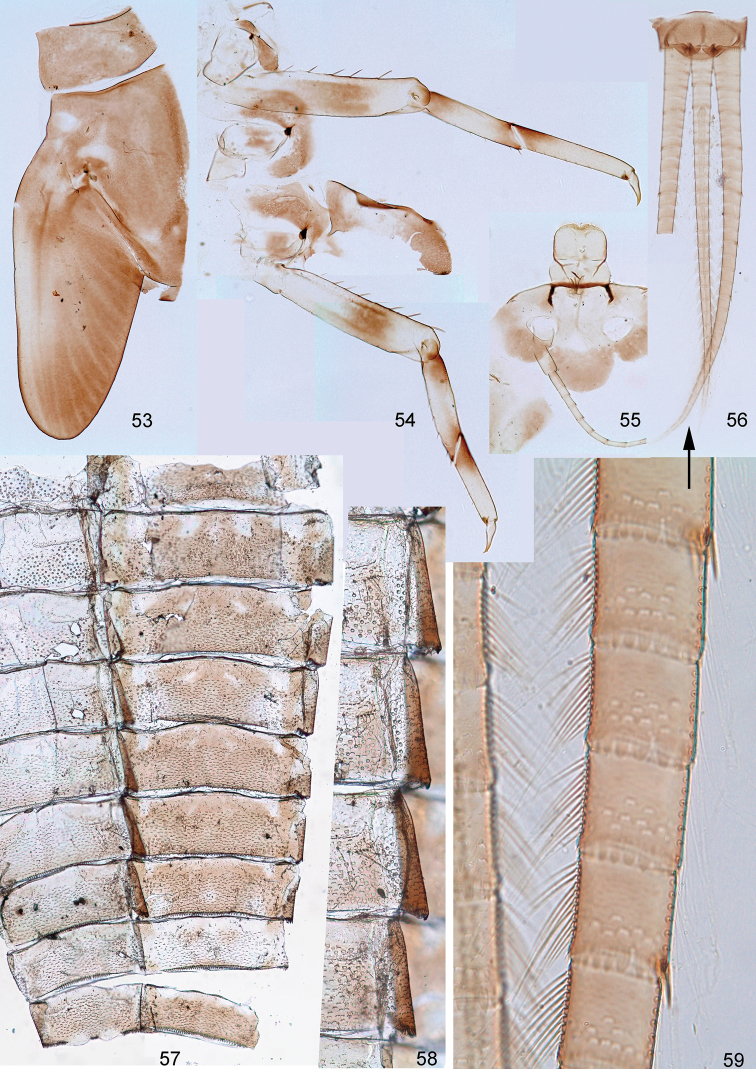
Larval exuviae of *Centroptella longisetosa* (neotype of *C. liebenauae*). **53–57** at the same magnification: (**53**) left half of pro- and mesonotum (**54**) thoracic pleura, left half of metanotum, fore- and hind legs (**55**) frons, antenna and labrum (**56**) tenth uromere and caudalii; (**57**) abdominal sterna and terga I–IX **58** margins of abdominal sterna IV–VII **59** cercus.

**Figures 60–76.**
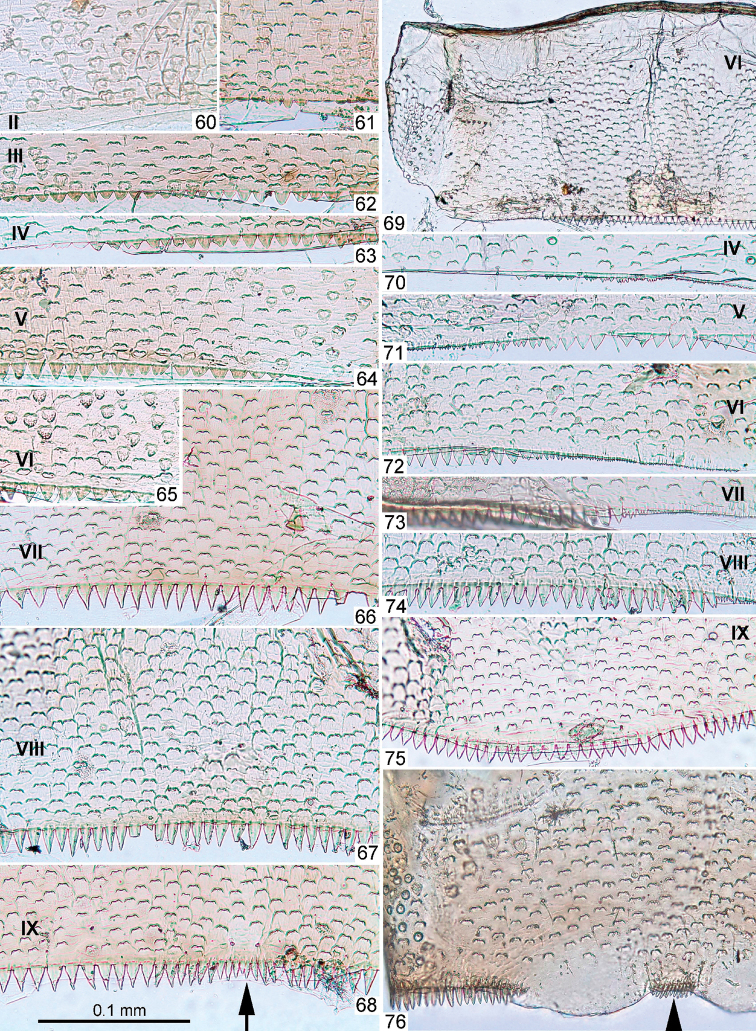
Larval exuviae of *Centroptella longisetosa*. **60–75** female, paratype of *C. longisetosa*: (**60**–**68**) fragments of abdominal terga II–IX (indicated by Roman numbers) (**69**) sternum VI (**70–75**) fragments of abdominal sterna IV–IX (indicated by Roman numbers) **76** male, neotype of *C. liebenauae*: fragment of abdominal sternum IX. Arrows on Figs 68 and 76 show median line.

**Figures 77–80.**
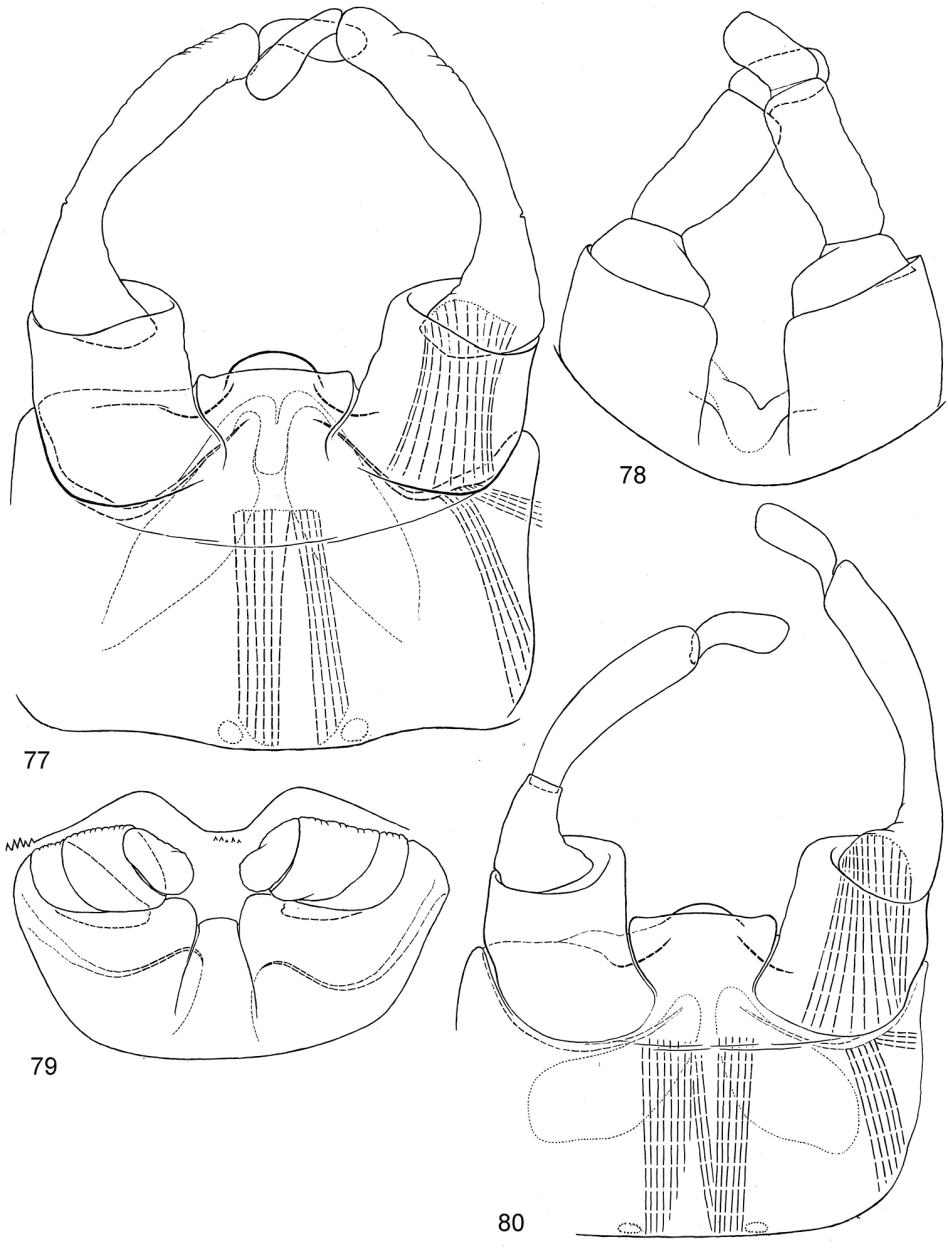
Male genitalia of *Centroptella longisetosa*. **77–79** specimens from India: (**77**) genitalia of imago (**78**) subimaginal exuviae (**79**) subimaginal gonostyli crumpled under larval cuticle (**80**) paratype of *C. liebenauae* (possibly reared from neotype): genitalia of imago.

**Figures 81–82.**
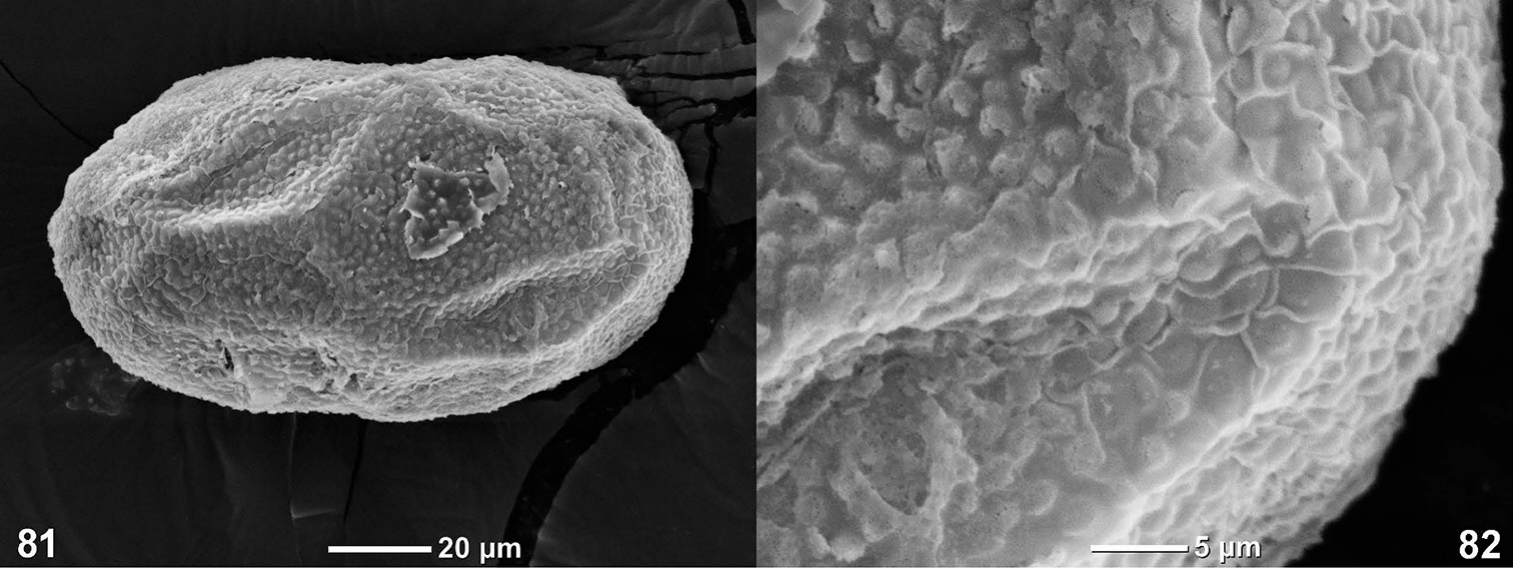
Egg extracted from larva – paratype of *Centroptella longisetosa*.

***Subimago.*** Adequately described by Soldán et al. (1987). Additional details: On all legs of male and female, all tarsal segments entirely covered with pointed microlepides (as in Fig. 137).

***Imago, male.*** Adequately described by Soldán et al. (1987). Additional details: Length of femur, tibia and tarsal segments (mm) on foreleg 0.8 : 1 : 0.04 : 0.55 : 0.4 : 0.21 : 0.12, on hind leg 0.55 : 0.54 : 0.15 : 0.09 : 0.04 : 0.1. Tarsus of middle and hind leg with one apical spine on initial 3^rd^ tarsomere (next after 1^st^+2^nd^ tarsomere) (as in Fig. 137). Genitalia: Figs 77, 80. Sterno-styligeral muscle present. Area between unistyligers forms well-outlined, trapezoid, colourless plate with distal margin widest; distal margin shallowly convex at middle and shallowly concave laterally, forming well-expressed angles adjacent to unistyligers. Gonostylus with 1^st^ segment short and conic; 2^nd^ segment thickened toward apex; 3^rd^ segment elongate, narrow and thickened toward apex, with proportions varying individually (Fig. 77). Penial bridge medially with semicircular, sclerotized, colourless projection and with pair of small, oblique, arched, sclerotized ridges proximad of it. Gonovectes shallowly bent, narrowed toward apices.

***Imago, female.*** Adequately described by Soldán et al. (1987). Additional details: Patella-tibial suture present on middle and hind legs, absent on forelegs (as in male). Tarsus of each leg with one apical spine on initial 3^rd^ tarsomere (on foreleg—on tarsomere next after 2^nd^ tarsomere, on middle and hind leg—on tarsomere next after 1^st^+2^nd^ tarsomere) (as in Fig. 136).

***Eggs*** (extracted from mature female larva, paratype of *C. longisetosa*). Oval, chorion with numerous irregular small protuberances (Figs 81, 82).

##### Dimension.

According to original descriptions, specimens from Vietnam (type series of *C. liebenauae*) smaller, 3.7–4.3 mm; specimen from China (type series of *C. longisetosa*) larger, 3.9–5.2 mm.

##### Variability.

All 8 examined specimens from India have maxillary palp relatively long (about 0.8 of lacinia length), while specimens from China and Vietnam have maxillary palp shorter (0.5–0.6 of lacinia length, Figs 46–48). In all other respects larvae from India have the same structure as larvae from China and Vietnam, and we were unable to find any differences between them, other than the size of maxillary palp. Imagines reared from larvae of the Indian form, are indistinguishable from imagines of the typical form, and have the same unusual styliger structure. Possibly, the examined specimens from India belong to a separate geographical form, which can be considered as a separate subspecies of the species *C. longisetosa*.

##### Remarks about descriptions and figures.

The original description of *C. longisetosa* contains some errors. Instead of foreleg (Braasch and Soldán 1980: fig. 2, “Vorderbein”), middle leg is shown, as evidenced by the presence of the patella-tibial suture (Fig. 50); the text also refers to the middle tibia (“Tibia ... wenig länger als der Tarsus”), while foretibia is shorter than tarsus (Fig. 54). Tergalius of first pair is wrongly figured (Braasch and Soldán 1980: fig. 5); probably, this drawing was made from tergalius of sixth pair (Fig. 30). Labrum (Braasch and Soldán 1980: fig. 8) has wrong shape and demonstrates posterior surface. Maxilla is wrongly drawn and characterized as “Maxille (Fig. 11) apikal dreizähnig”; actually, it has four apical denticles, if one regards the first dentiseta to be one of these denticles (Fig. 42).

Waltz and McCafferty (1987b) examined the paratype of *C. longisetosa* (see above) and wrote that “The secondary row of tibial setae as illustrated for *C. soldani* (figs 3i and j of Müller-Liebenau 1983) is not present in *C. longisetosus* contrary to the data indicated in table 2 of Müller-Liebenau 1983)”. Probably, under the “secondary row” they mean that arm of the U-shaped tibial row, which is located on the posterior side of the tibia; this posterior arm is present in *C. longisetosa* (Figs 49–51), as well as in all other *Centroptella*.

Tong et al. (2003) redescribed the larva of *C. longisetosa* and described its imagines. Their figures, including figures of tergalii III, V and VII agree well with the species described here, but tergalius I is figured incorrectly with a rounded apex (Tong et al. 2003: fig. 8).

The larval metanotum is figured by Tong et al. (2003: fig. 7) with a vestige of hind protopteron; based on larvae from the same series, Salles et al. (2016: fig. 6K) figured it without these vestiges. In all eight larvae of the last instar examined by us, including a paratype of *C. longisetosa* and the neotype of *C. liebenauae*, vestiges of hind protoptera are present (Fig. 54).

On the figure of male imaginal genitalia (Tong et al. 2003: fig. 14), the trapezoid plate between unistyligers is correctly figured ventrad of the semicircular penial projection, but on the figure by Salles et al. (2016: fig. 9C), made from a specimen of the same series, this trapezoid plate is wrongly figured dorsad of the semicircular projection.

#### Centroptella ingridae sp. nov.

Taxon classificationAnimaliaEphemeropteraBaetidae

797555B0-2517-533F-872A-2993D0A8C6C1

http://zoobank.org/0D1B2BEB-824D-4881-B219-29635BBB5C1D

Figures 33–39 , 83–89 , 90–93 , 94–109 , 110–115 , 116–122 , 123–128 , 129–132 , 133–137 , 138–143 , 144–148 , 149

Chopralla sp.: Waltz and McCafferty 1987a: 183 (larva in list of material examined)
Centroptella liebenauae : Soldán et al. 1987: 342 (partim: larva, non imago)
Bungona (Chopralla) liebenauae : Salles et al. 2016: 104, fig. 6D (larva), Appendix S3 (partim: larval characters 0–19 and 31–121); Sroka et al. 2019: fig. 6B (larva); Shi and Tong 2019: 582, figs 60–67 (larva)

##### Material examined.

***Holotype***: L-S-I♂ {specimen [XV](1)2015}, THAILAND, Mae-Hong-Son Province, Pai, Mhor-Phaeng Falls, 11.II.2015, coll. N. Kluge & L. Sheyko (ZIN). ***Paratypes***: the same locality and collectors, 9–11.II.2015: 1 L-S♂, 2 L-S-I♀, 2 L/S♂, 6 larvae (ZIN); Pai, 19–25.XI.2010, coll. K. Tomkovich: 1 I♂ (ZIN). VIETNAM, Vinn Phu Prov., Suoi Bac Stream, Tam-Dao, 10–16.10.1984 T. Soldán: 47 larvae (paratypes of *Centroptella liebenauae*, including the specimen wrongly labeled as “holotype”, see above) (deposited in Institute of Entomology, BC CAS, České Budějovice).

##### Etymology.

This species is named in honour of Ingrid Müller-Liebenau.

##### Descriptions.

**Figures 83–89.**
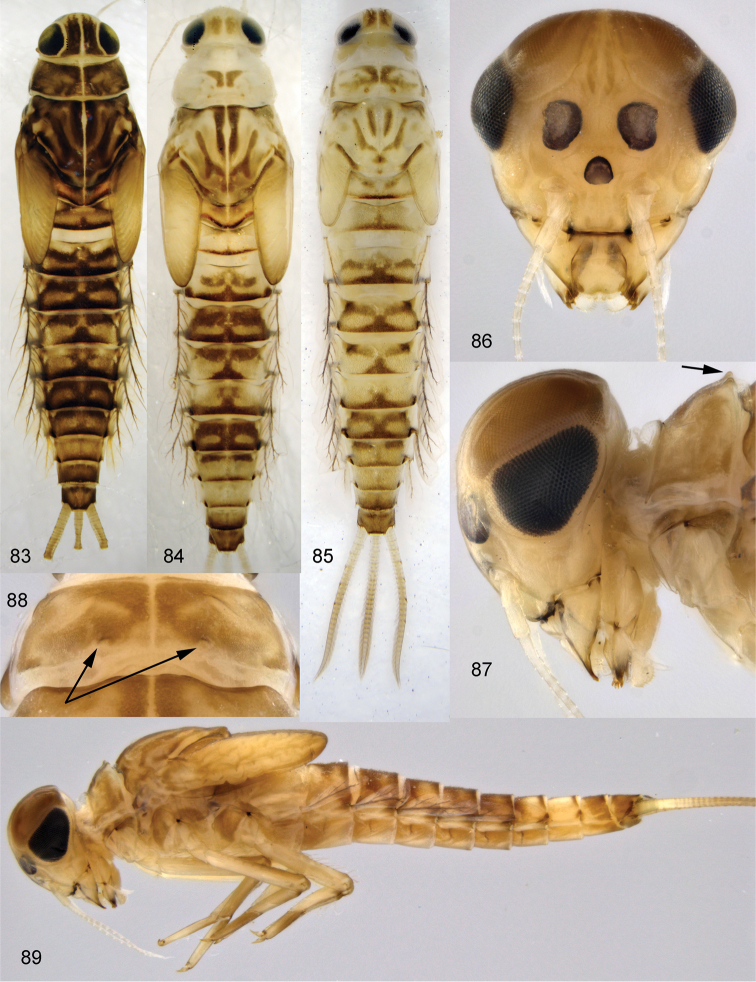
*Centroptella ingridae* sp. nov., larvae. **83–85** specimens from Thailand **86–89** paratypes of *C. liebenauae* (actually *C. ingridae* sp. nov.). Arrows show pair of protuberances on pronotum.

***Larva*.***Cuticular coloration*. Frontal side of head colourless (Fig. 86). Pronotum and mesonotum with contrasting brown, ochre and/or colourless areas, forming characteristic pattern (Figs 83–85). Thoracic pleura and metanotum partly brown, partly colourless; sterna colourless (Fig. 89). Each leg with coxa and trochanter colourless; femur either entirely colourless, or with diffuse brown macula in distal part on posterior and/or anterior surfaces; tibia and tarsus with more or less expressed diffuse brown coloration, mainly on outer side; claws brownish (Fig. 89). Abdominal terga with contrasting brown, ochre and/or colourless, areas forming characteristic pattern; most terga with large, paired, transverse blanks, which occupy medioposterior sigilla and stretch laterally from them (Figs 83–85). Caudalii colourless at base, diffusely darkened at middle (Fig. 85).

*Shape and setation*. Frontal suture short, nearly semicircular (as in Fig. 55). Labrum equally wide at base and middle, with pair of submedian, long setae, 2–3 pairs of sublateral, long setae and pair of long setae between submedian and sublateral ones (Fig. 116). Prostheca of left mandible with 3 blunt processes and 2–4 pointed processes (Fig. 144). Prostheca of right mandible directed medially-distally, with short, apical denticles and without long branch; median margin of right mandible proximad of prostheca either without processes, or with small seta-like processes (Fig. 145). Maxillary canines and distal dentiseta stout; distal dentiseta widened, with apex somewhat hooked toward canines (as in Fig. 42). Maxillary palp nearly as long as lacinia, 2-segmented. Labium with glossae and paraglossae subequal, both narrowed apically (Fig. 124). Glossa ventrally with irregularly arranged setae in proximal part and about 10 setae forming ventro-median row. Paraglossa with latero-apical setae forming one regular row and few setae just dorsad of it; with about 8 setae in ventro-median row; with 4 setae in dorso-median row. Distal segment of labial palp rounded apically (Fig. 123).

**Figures 90–93.**
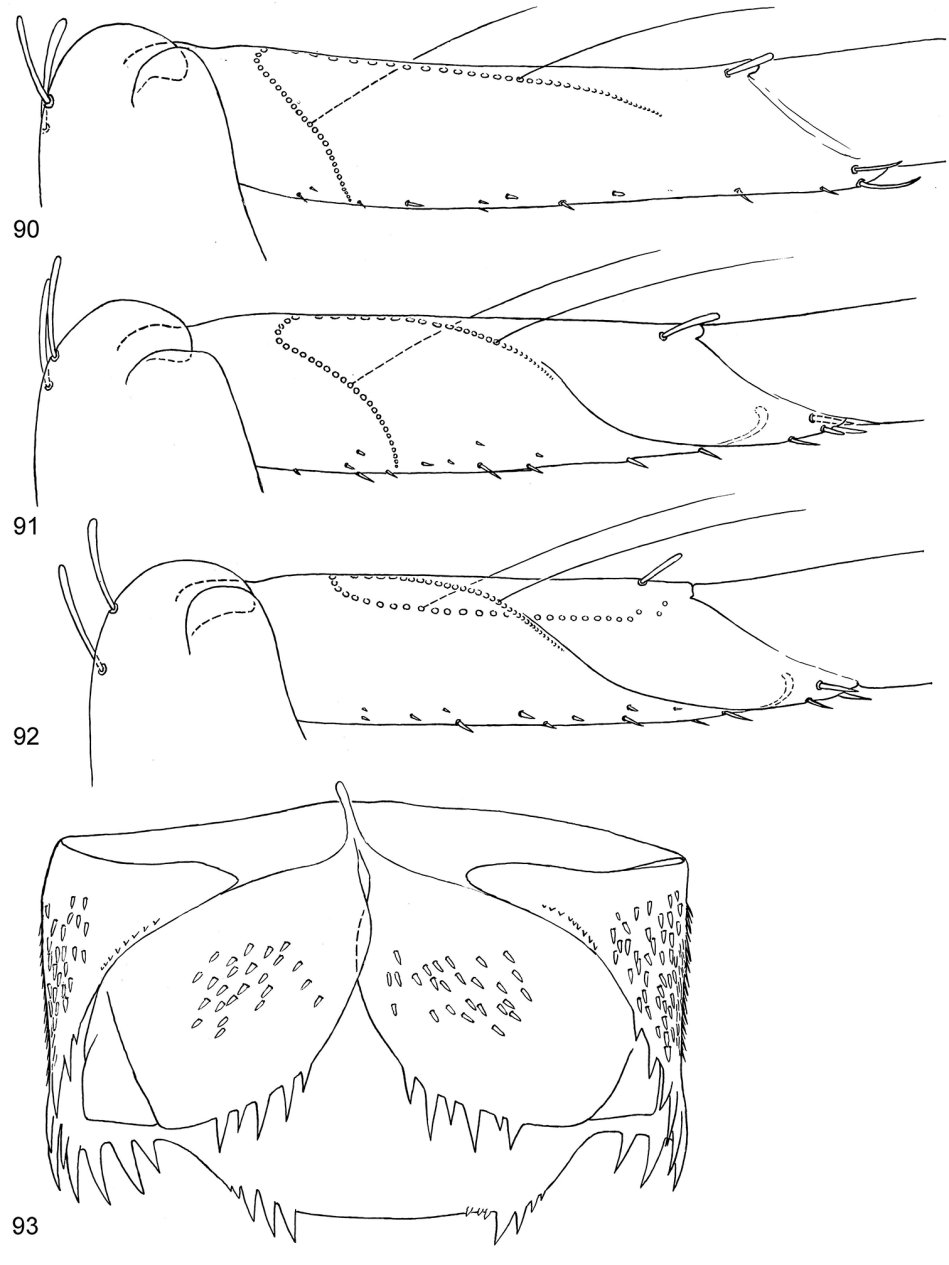
*Centroptella ingridae* sp. nov. (holotype). **90–92** tibia of fore, middle and hind leg, view from anterior side (bases of long setae shown both on anterior and posterior sides) **93** tenth abdominal segment without caudalii, ventral view.

Pronotum with pair of protuberances near posterior margin (Figs 87, 88, 111; the same character listed by Shi and Tong 2019: 583, figs 62, 63 under species name *C. liebenauae*). Metanotum with vestiges of hind protoptera (as in Fig. 54). Forelegs longest, hind legs shortest; on all legs, tarsus (measured on outer side) longer than tibia; in holotype length of femur / tibia / tarsus / claw of foreleg (mm) 0.88 : 0.48 : 0.57 : 0.16; on middle leg 0.83 : 0.42 : 0.48 : 0.16; on hind leg 0.80 : 0.39 : 0.43 : 0.16. Femur parallel-sided; outer margin straight or slightly concave, apically either rounded (Figs 90–92), or with blunt-angled projection bearing two subapical setae; inner margin slightly convex. Outer side of femur with regular or irregular row of 9–11 long, blunt setae and 2 subapical setae of same form (Figs 90–92). Inner-dorsal side of forefemur with few stout setae, length of these setae being half that of setae on dorsal side. Foreleg without patella-tibial suture, middle and hind legs with patella-tibial suture greatly shifted to apex of tibia. Posterior arm of U-shaped row of long setae on fore- and middle leg situated across tibia (Figs 90–91); on hind leg longitudinal (Fig. 92). Inner margin of tibia and tarsus with irregular, small, stout, pointed setae. Outer-apical seta of tibia blunt and elongate (Figs 90–92). Dorsal side of each tarsus with long, fine setae situated irregularly and partly forming two longitudinal rows. Claw either with two rows of denticles (Fig. 127) or with their vestiges (Fig. 126).

**Figures 94–109.**
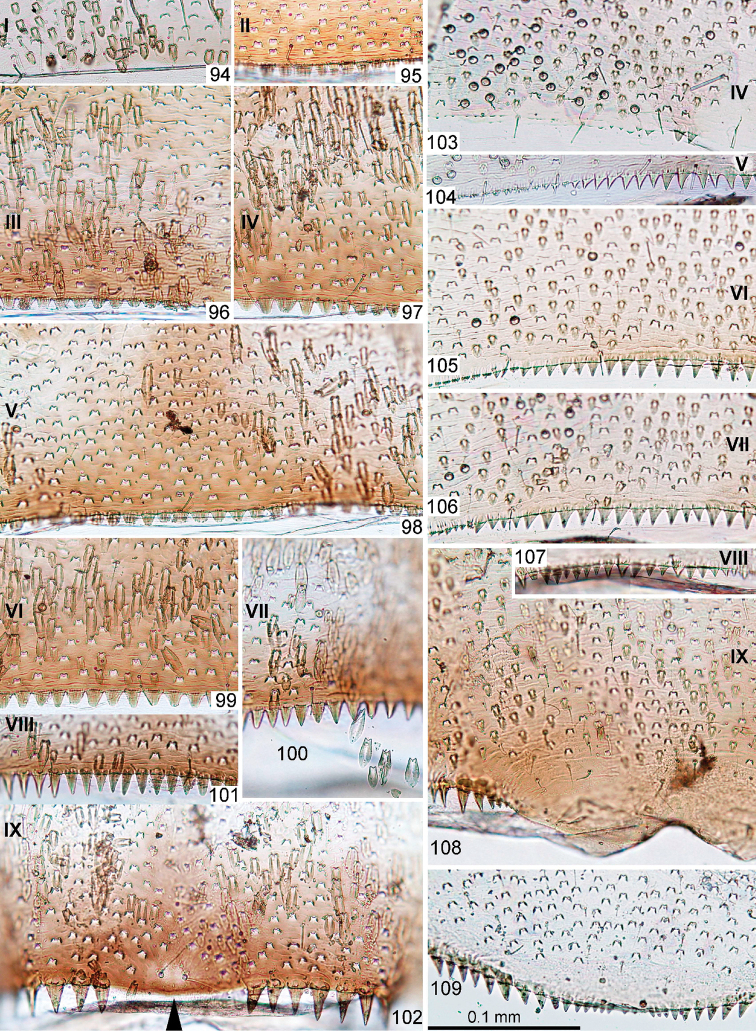
*Centroptella ingridae* sp. nov. **94–102** fragments of abdominal terga I–IX (indicated by Roman numbers) **103–108** fragments of abdominal sterna IV–IX of male (indicated by Roman numbers) **109** fragment of abdominal sternum IX of female (**94–101** and **103–108** male paratype of *C. liebenauae*; 102 and 109 female specimen from Thailand).

**Figures 110–115.**
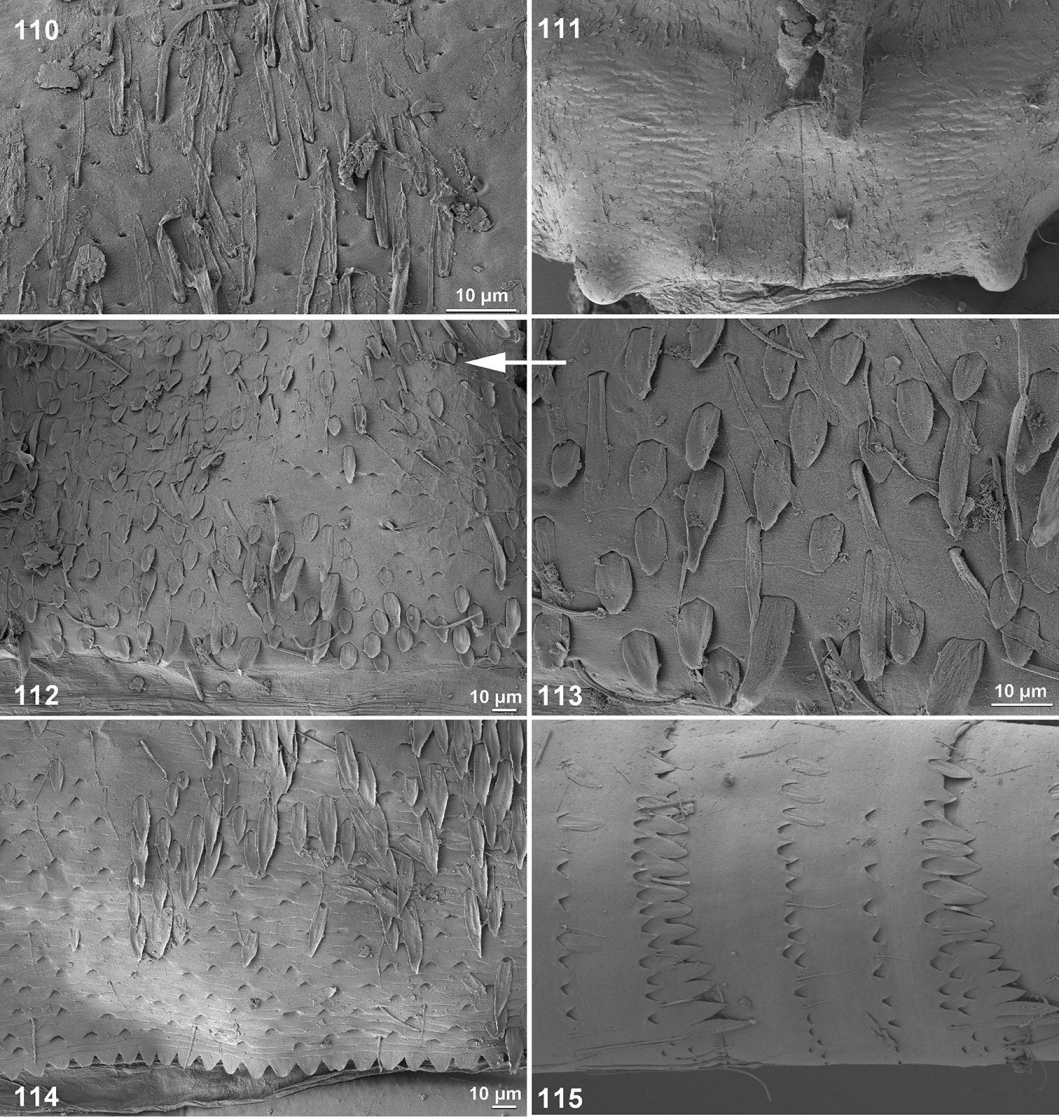
*Centroptella ingridae* sp. nov. (paratype of *C. liebenauae*), SEM photos of larva. **110** clypeus **111** pronotum **112–113** abdominal tergum I **114** abdominal tergum III **115** cercus.

Scales on abdominal terga and sterna numerous, elongate, varying in size and shape, bordered by brown (Figs 94–122). Posterior margin of abdominal tergum I smooth, without denticles (Fig. 94); posterior margins of terga II–VI with short semicircular and triangular denticles (Figs 95–99); terga VII–IX with longer triangular denticles (Figs 100–102); on tergum IX middle part of hind margin behind pair of submedian setae lack denticles and projected posteriorly (Fig. 102). Posterior margin of tergum X without denticles on median part, laterally with paired groups of several denticles, decreasing in length in lateral direction (Fig. 93). Posterior margins of abdominal sterna I–IV smooth (Fig. 103); posterior margins of sterna V–VIII with regular, small, pointed, triangular denticles (Figs 104–107). Posterior margin of sternum IX in female convex, with row of triangular denticles diminished medially (Fig. 109), in male without denticles between protogonostyli, but with several denticles at sides (Figs 108, 148). Each sternum IV–VI with pair of regular, transverse rows of long, fine, bifurcate setae with spaced sockets (Figs 117–119); other sterna either without such setae, or with few, smaller setae irregularly situated. Paraprocts with small, anterior, median apodeme, with few large pointed denticles on posterior margin, with scales as on sterna and terga (Figs 93, 120–121). Tergalius I narrow, elongate-ellipsoid; other tergalii wider, gradually changing in shape from tergalius II to tergalius VII (Figs 33–39). Each tergalius II–VII, besides costal and anal ribs, with straight and narrow middle rib, located on dorsal surface on background of main trachea (Fig. 125). Costal margin with poorly expressed serration; anal margin without serration; outer margin free of ribs, slightly notched, with small seta in each notch. In middle part of cercus, lateral side with 2 long, pointed denticles on every 4^th^ segment (Figs 129–130). Each cercus, besides regular row of primary swimming setae on inner side (Fig. 132), with smaller and thinner secondary swimming setae on outer margin; on distal half of cercus, secondary swimming setae with wide, transverse, oval bases, forming regular row (Figs 129–131); on proximal half of cercus, secondary swimming setae with small, round bases and situated irregularly (Figs 129–130).

*Male genitalia*. In last larval instar, developing subimaginal gonostyli folded under larval cuticle in peculiar pose, with 3^rd^ segments bent medially-proximally (Fig. 148).

**Figures 116–122.**
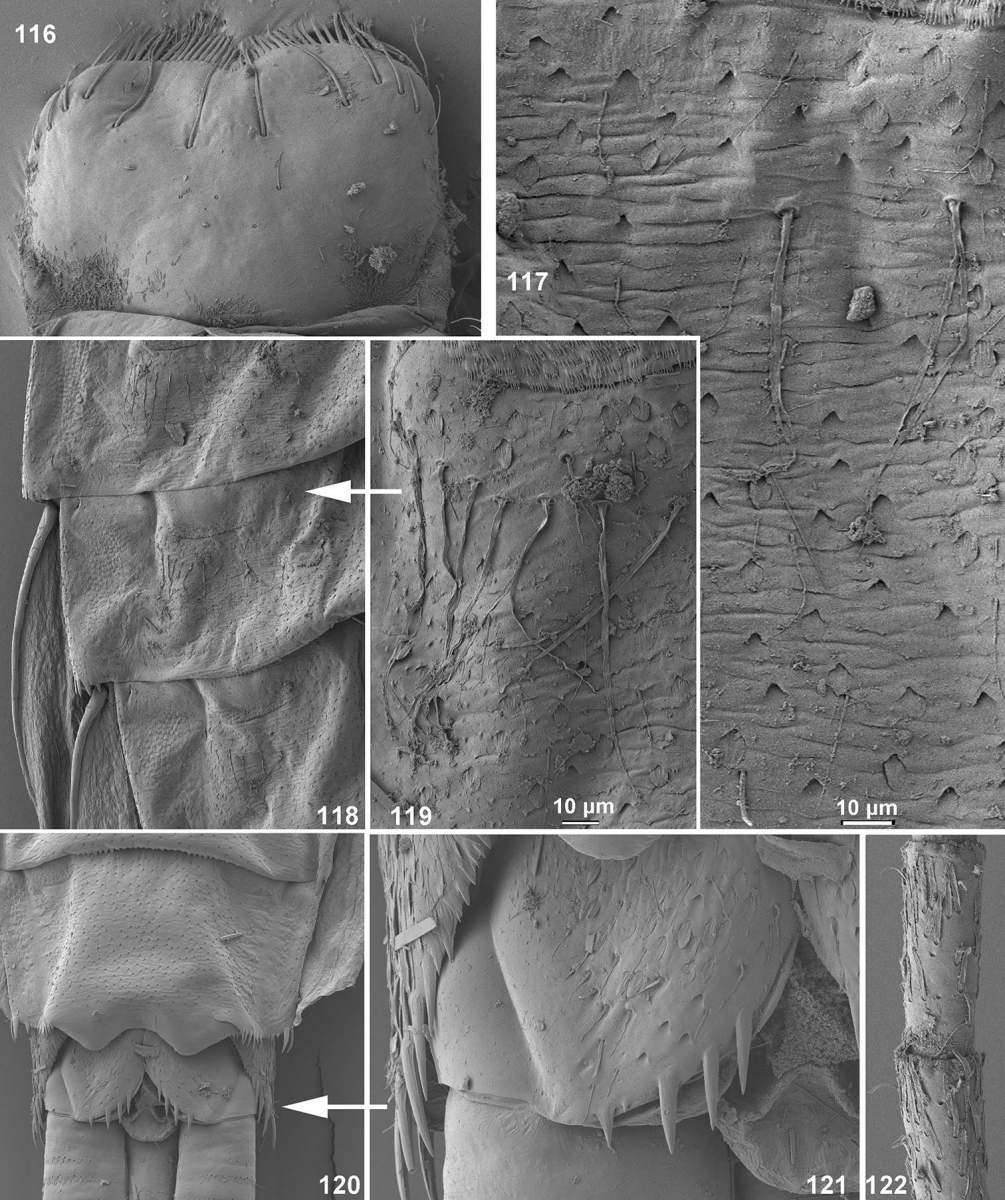
*Centroptella ingridae* sp. nov. (paratype of *C. liebenauae*), SEM photos of larva. **116** labrum **117** bifurcate setae on abdominal sternum IV **118–119** abdominal sterna IV–VI **120–121** paraprocts **122** flagellum of antenna.

**Figures 123–128.**
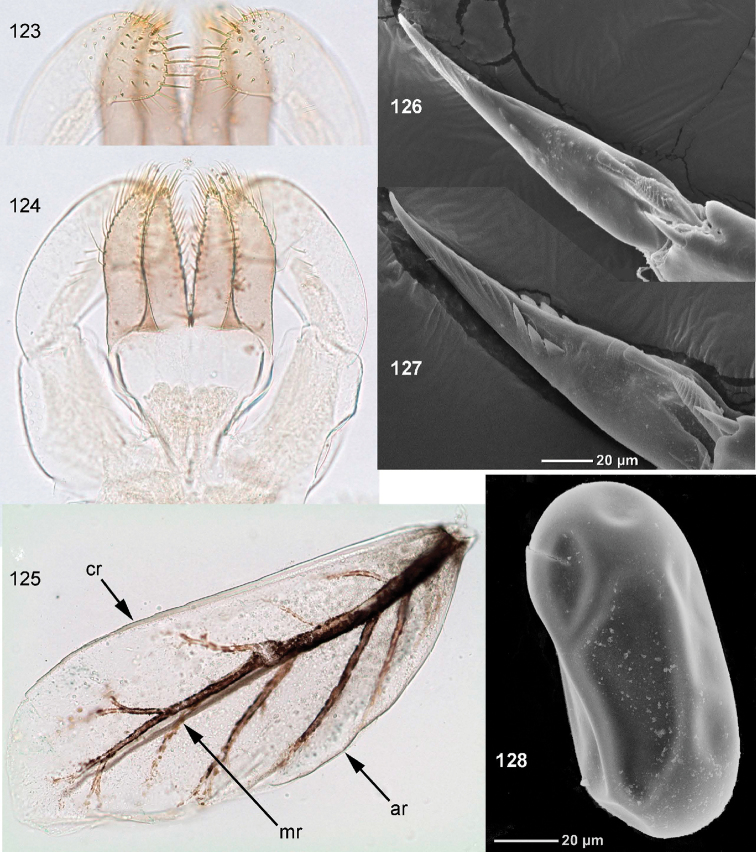
*Centroptella ingridae* sp. nov. (specimens from Thailand). **123–124** labium **125** tergalius IV (**cr** costal rib; **mr** middle rib; **ar** anal rib) **126–127** larval claws; **128** egg.

**Figures 129–132.**
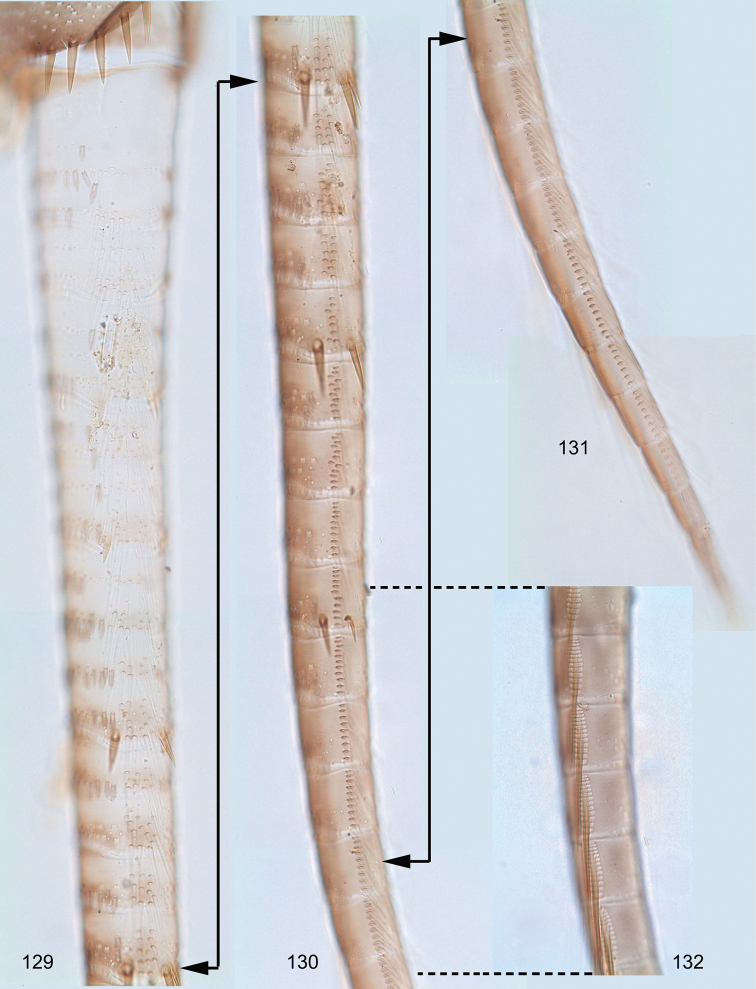
*Centroptella ingridae***sp. nov.** (specimen from Thailand), exuviae of larval cercus (lateral view). **129–131** focus on lateral side to show bases of secondary swimming setae; **132** focus on median side to show bases of primary swimming setae.

**Figures 133–137.**
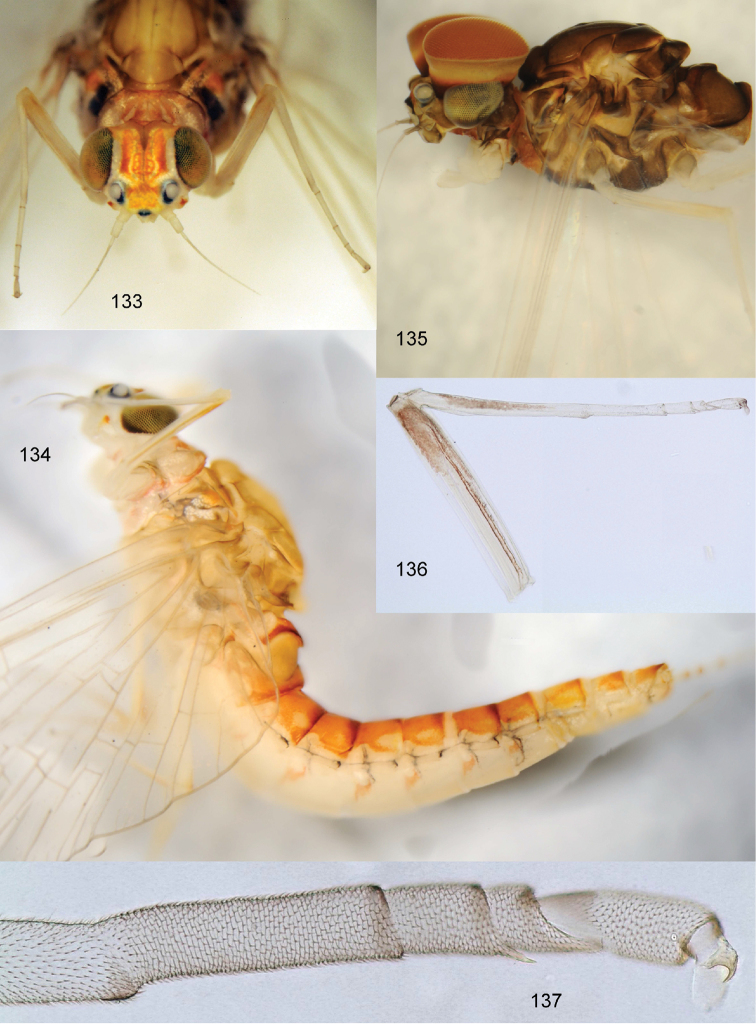
*Centroptella ingridae* sp. nov. (specimens from Thailand). **133–134** female imago **135–137** holotype (male): (**135**) head and thorax of imago (**136**) imaginal middle leg (**137**) subimaginal exuviae of tarsus.

**Figures 138–143.**
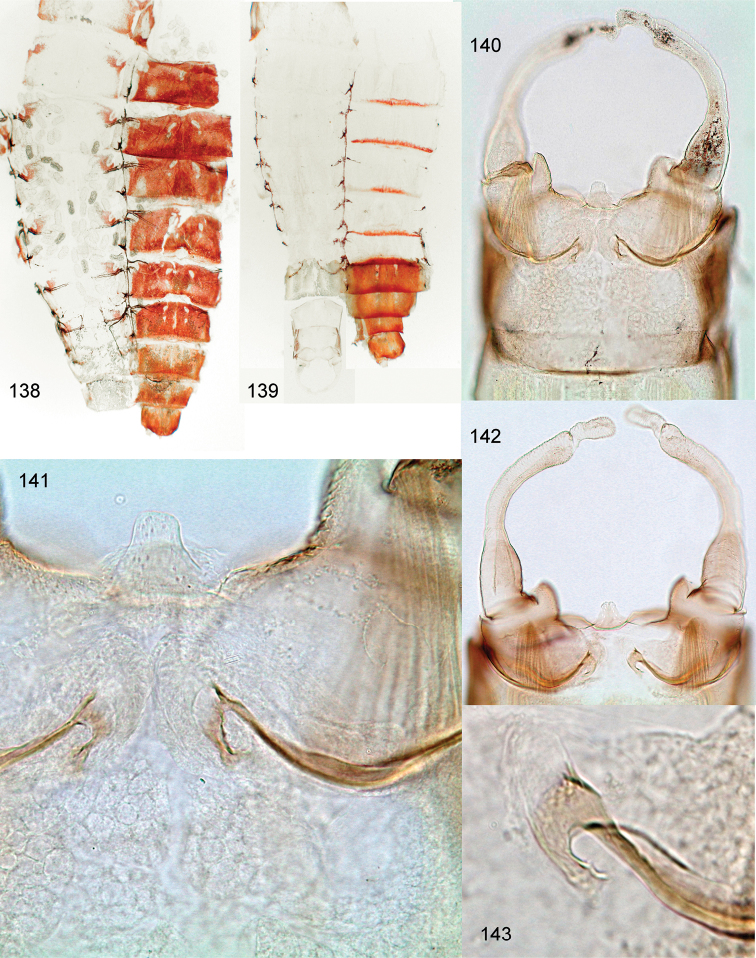
*Centroptella ingridae* sp. nov. (specimens from Thailand). **138** abdominal sterna and terga of female imago **139** the same, male imago **140–143** genitalia of male imago (**139–141** holotype).

**Figures 144–148.**
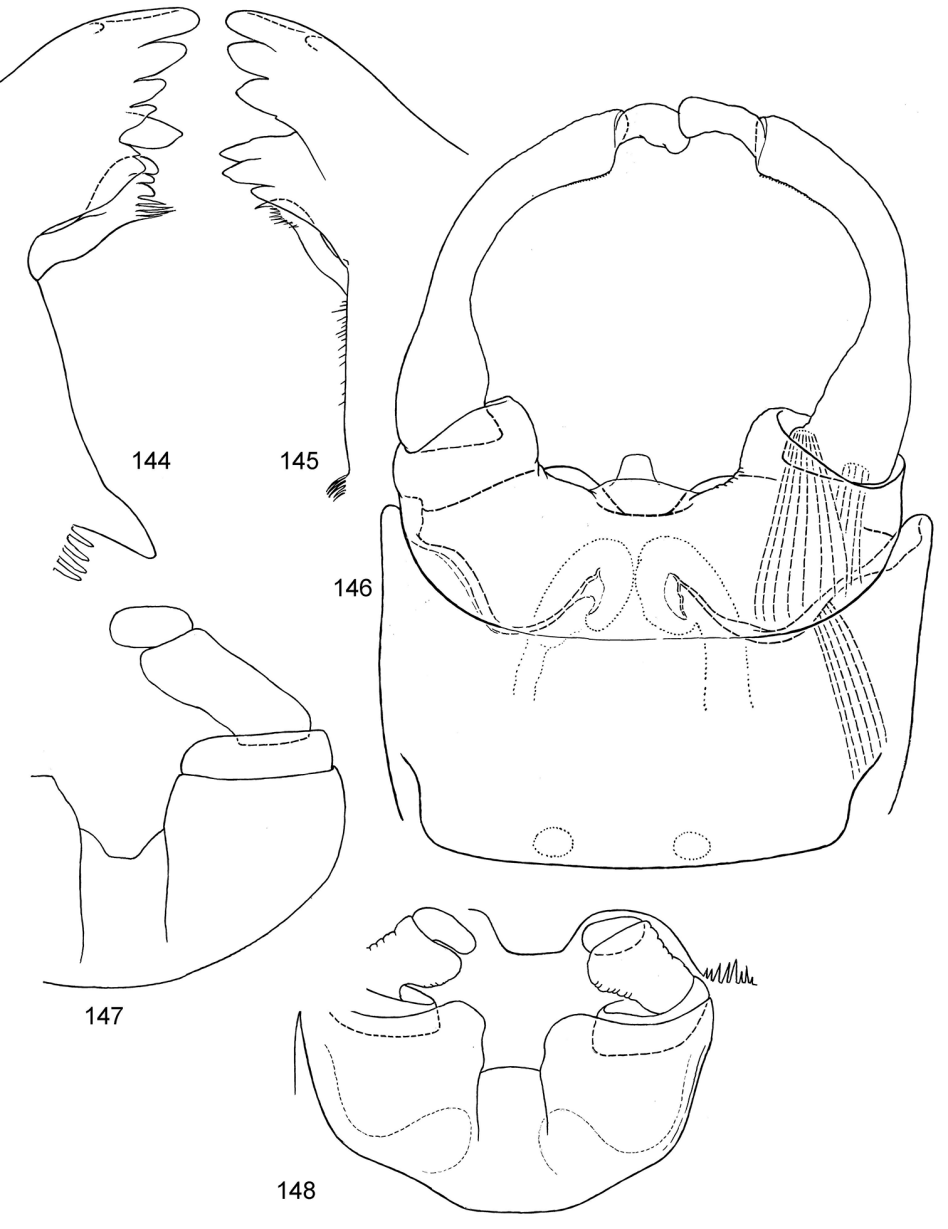
*Centroptella ingridae* sp. nov. (specimens from Thailand). **144–145** left and right mandibles **146** genitalia of male imago **147** their subimaginal exuviae **148** subimaginal gonostyli crumpled under larval cuticle (146–147 holotype).

**Figures 149–152.**
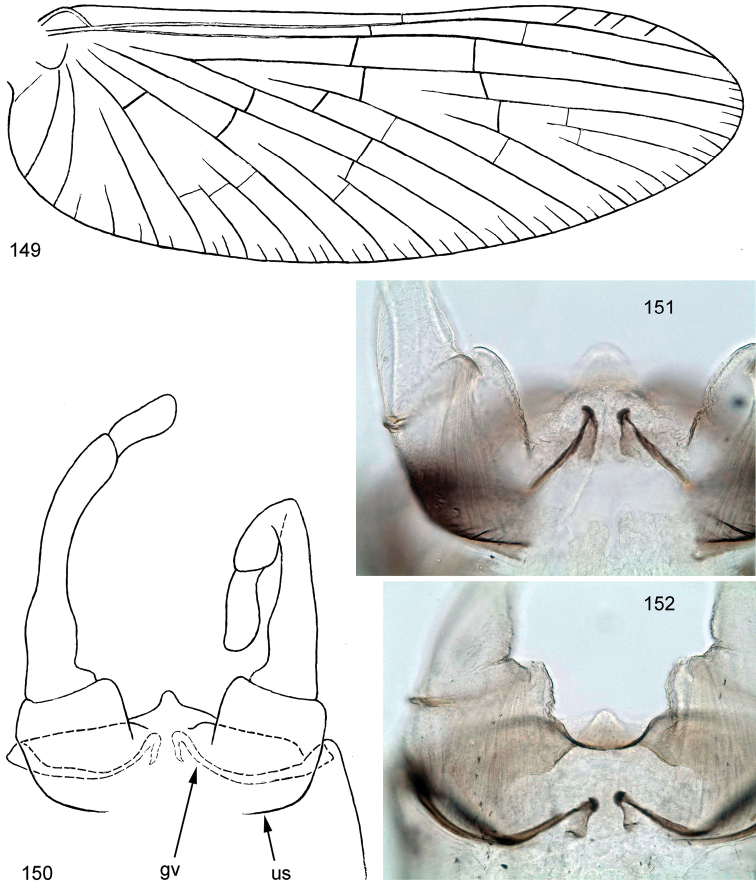
*Centroptella* of group *inzingae-ingridae*. *Centroptella ingridae* sp. nov. (holotype), fore wing **150***Centroptella inzingae* (lectotype), genitalia (**gv** gonovectis **us** proximal margin of unistyliger) **151–152***Centroptella saxophila*, gonovectes (specimens reared from larvae by N. Kluge in the Western Cape Province of South Africa).

***Subimago.*** Cuticle light brown with darker brown sutures of thorax; hypodermal coloration as in imago. On all legs of male and female all tarsal segments entirely covered by pointed microlepides (Fig. 137).

***Imago, male.*** Head brown. Turbinate eyes relatively low and wide, with yellow stem and orange-red facetted surface (Fig. 135). Thorax dark brown, with ochre pleural membranes (Fig. 135). Wing (Fig. 149) with membrane colourless, veins pale ochre or colourless, extreme base of costal and subcostal veins proximad of costal brace brown. Femora of all legs ochre, apically diffusely tinged with reddish; foretibia light ochre, apically darkened with light brownish; middle and hind tibiae ochre, with diffuse longitudinal stripe; tarsi of all legs pale ochre; claws brown (Fig. 136). In holotype, length of femur, tibia and tarsal segments (mm) on foreleg 1.05 : 1.13 : 0.05 : 0.55 : 0.35 : 0.19 : 0.15, on middle leg 0.78 : 0.62 : 0.25 : 0.09 : 0.05 : 014, on hind leg 0.74 : 0.57 : 0.21 : 0.07 : 0.04 : 0.14. Tarsus of middle and hind leg with 1 apical spine on initial 3^rd^ tarsomere (next after 1^st^+2^nd^ tarsomere) (as in Fig. 137). Abdominal tergum I colourless; terga II–VI colourless with narrow, contrasting, reddish stripe bordering posterior margin; terga VII–X red with ochre, with darker stripe bordering posterior margin; abdominal sterna colourless (Fig. 139). Genitalia (Figs 140–143, 146). Sterno-styligeral muscle entirely absent. Posterior margin of 9^th^ abdominal sternum between unistyligers with narrow, trapezoid, membranous, colourless process (Figs 141, 146). Gonostylus with 1^st^ segment narrowed apically; 2^nd^ segment thickened toward apex; 3^rd^ segment elongate, narrow and thickened toward apex (Figs 142, 146). Penial bridge medially sharply concave (Fig. 146). Gonovectes apically with sclerotized widenings of peculiar halberd-like shape (Figs 141, 143).

***Imago, female.*** Head and thorax ochre with reddish markings (Figs 133–134). Leg coloration as in male. Patella-tibial suture present on middle and hind legs, absent on forelegs (as in male). Tarsus of each leg with 1 apical spine on initial 3^rd^ tarsomere (on foreleg – on tarsomere next after 2^nd^ tarsomere, on middle and hind leg—on tarsomere next after 1^st^+2^nd^ tarsomere) (as in Fig. 137). Abdominal terga intensely coloured with ochre and reddish, partly repeating cuticular colour pattern of larva; abdominal sterna nearly colourless, sterna I–VI with pair of reddish maculae near antero-lateral corners (Fig. 138).

***Egg***. Oval; chorion smooth, without relief (Fig. 128).

##### Dimension.

Forewing length of male 4.7 mm; of female 5.0 mm.

##### Distribution.

Indochina: known from Thailand and Vietnam; recently reported from China (Yunnan, Guangxi, Guangdong) under the species name *C. liebenauae* (Shi and Tong 2019).

##### Comparison.

*Centroptella ingridae* sp. nov. belongs to the *inzingae-ingridae* species group; male imagines of this group differ from all other *Centroptella* by halberd-like gonovectes and absence of the sterno-styligeral muscle (Fig. 146). The male imago of *Centroptella ingridae* sp. nov. differs from other members of the *inzingae*-*ingridae* group by abdominal coloration (Figs 139). The larva of *Centroptella ingridae* sp. nov. differs from all other *Centroptella* by the presence of a pair of projections on the pronotum (Fig. 111).

#### Centroptella colorata

Taxon classificationAnimaliaEphemeropteraBaetidae

Soldán, Braasch & Muu, 1987

8819B368-1AB2-5884-B5D0-DF808913432B

Figure 153

Centroptella colorata Soldán, Braasch & Muu, 1987: 346 (larva)
Chopralla colorata : Tong and Dudgeon 2003: 17 (larval generic characters)
Bungona (Chopralla) colorata : Salles et al. 2016: 104; Shi and Tong 2019: 581

##### Material examined.

***Holotype***: male larva of last instar, without caudalii, with labels: “VIETNAM, Lam Dong Prov, Da Nhim riv., Duc Trong, 27.X.1984, T. Soldán”, “*Centroptella colorata* T. Soldán det. 1985” and “HOLOTYPE”. Paratypes not found.

**Figure 153.**
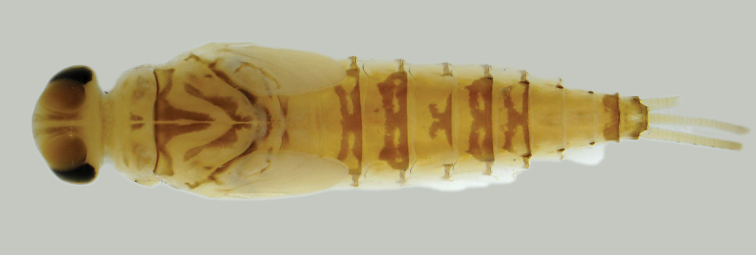
*Centroptella colorata* (holotype), last instar larva.

##### Additional characters.

Abdominal tergum IV without denticles on posterior margin, so regular row of denticles present on posterior margin of terga V–IX only. Tergum X without denticles on median part of posterior margin, with one pair of large denticles at sides (as in *C. ceylonensis*).

## Supplementary Material

XML Treatment for Centroptella

XML Treatment for Centroptella longisetosa

XML Treatment for Centroptella ingridae

XML Treatment for Centroptella colorata
